# Impaired function of rDNA transcription initiation machinery leads to derepression of ribosomal genes with insertions of R2 retrotransposon

**DOI:** 10.1093/nar/gkab1276

**Published:** 2022-01-17

**Authors:** Elena A Fefelova, Irina M Pleshakova, Elena A Mikhaleva, Sergei A Pirogov, Valentin A Poltorachenko, Yuri A Abramov, Daniil D Romashin, Aleksei S Shatskikh, Roman S Blokh, Vladimir A Gvozdev, Mikhail S Klenov

**Affiliations:** Department of Molecular Genetics of the Cell, Institute of Molecular Genetics of National Research Centre «Kurchatov Institute», Moscow 123182, Russia; Division of Biology and Biological Engineering, California Institute of Technology, Pasadena 91125, USA; Department of Molecular Genetics of the Cell, Institute of Molecular Genetics of National Research Centre «Kurchatov Institute», Moscow 123182, Russia; Laboratory for Neurobiology of Memory, P.K. Anokhin Institute of Normal Physiology, Moscow 125315, Russia; Department of Molecular Genetics of the Cell, Institute of Molecular Genetics of National Research Centre «Kurchatov Institute», Moscow 123182, Russia; Department of Molecular Genetics of the Cell, Institute of Molecular Genetics of National Research Centre «Kurchatov Institute», Moscow 123182, Russia; Department of Molecular Genetics of the Cell, Institute of Molecular Genetics of National Research Centre «Kurchatov Institute», Moscow 123182, Russia; Department of Molecular Genetics of the Cell, Institute of Molecular Genetics of National Research Centre «Kurchatov Institute», Moscow 123182, Russia; Laboratory of Precision Biosystems, V. N. Orekhovich Institute of Biomedical Chemistry, 10 Pogodinskaya St., Moscow 119121, Russia; Department of Molecular Genetics of the Cell, Institute of Molecular Genetics of National Research Centre «Kurchatov Institute», Moscow 123182, Russia; Department of Molecular Genetics of the Cell, Institute of Molecular Genetics of National Research Centre «Kurchatov Institute», Moscow 123182, Russia; Department of Functional Genomics, Institute of Gene Biology, Russian Academy of Sciences, 34/5 Vavilova Street, Moscow 119334, Russia; Department of Molecular Genetics of the Cell, Institute of Molecular Genetics of National Research Centre «Kurchatov Institute», Moscow 123182, Russia; Department of Molecular Genetics of the Cell, Institute of Molecular Genetics of National Research Centre «Kurchatov Institute», Moscow 123182, Russia

## Abstract

Eukaryotic genomes harbor hundreds of rRNA genes, many of which are transcriptionally silent. However, little is known about selective regulation of individual rDNA units. In *Drosophila melanogaster*, some rDNA repeats contain insertions of the R2 retrotransposon, which is capable to be transcribed only as part of pre-rRNA molecules. rDNA units with R2 insertions are usually inactivated, although R2 expression may be beneficial in cells with decreased rDNA copy number. Here we found that R2-inserted rDNA units are enriched with HP1a and H3K9me3 repressive mark, whereas disruption of the heterochromatin components slightly affects their silencing in ovarian germ cells. Surprisingly, we observed a dramatic upregulation of R2-inserted rRNA genes in ovaries lacking Udd (Under-developed) or other subunits (TAF1b and TAF1c-like) of the SL1-like complex, which is homologues to mammalian Selective factor 1 (SL1) involved in rDNA transcription initiation. Derepression of rRNA genes with R2 insertions was accompanied by a reduction of H3K9me3 and HP1a enrichment. We suggest that the impairment of the SL1-like complex affects a mechanism of selective activation of intact rDNA units which competes with heterochromatin formation. We also propose that R2 derepression may serve as an adaptive response to compromised rRNA synthesis.

## INTRODUCTION

Regulation of ribosomal DNA (rDNA) transcription is a fine-tuned mechanism that defines the global level of protein synthesis and controls cell growth and differentiation (1,2). In eukaryotes, production of rRNA by RNA polymerase I (Pol I) occurs in the nucleolus accounting for up to 60% of total transcriptional activity of the cell (3,4). Eukaryotic rDNA is organized in clusters also known as the nucleolus organizer regions, NORs, generally consisting of hundreds of tandemly repeated rRNA genes separated by intergenic spacer regions (IGS) (5). Each gene expresses a pre-rRNA transcript harboring an external transcribed spacer (ETS) followed by the sequences of 18S, 5.8S and 28S rRNAs, which are interspaced by internal transcribed spacers (ITSs). Both ETS and ITSs are eliminated during nuclease processing steps, leading to formation of mature rRNAs.

Numerous genetic, microscopic and biochemical studies have demonstrated that only a part of rDNA units is transcriptionally active at any time, while the rest, or even the majority of rDNA repeats, are in a repressed state (6–14). In organisms whose genomes contain several rDNA clusters, silencing can occur at the level of entire NORs, which is well described in plants, mammals and the fruit fly (15–18). Moreover, within the active NORs, some rRNA genes can be silent (19,20). For example, the mosaic clustering of active hypomethylated and repressed hypermethylated rDNA units was revealed in human cells (20). Repression of a fraction of rRNA genes can perform various functions. First, this phenomenon can be useful to maintain an optimal level of ribosome production regardless of the rDNA copy number in the genome (21). Second, maintaining a subset of rRNA genes in an inactive heterochromatic state is thought to be important for ensuring nucleolar structure and preventing recombination between rDNA repeats (22–24). Besides, it is necessary to repress defective rDNA units, which are abundant in eukaryotic genomes. For instance, approximately 20–30% of rDNA repeats in human cell lines are palindromic and noncanonical (20,25). Repressive histone modifications, DNA methylation and nucleosome remodeling have been identified as the mechanisms responsible for rDNA silencing in studies performed mainly in yeast and mammalian cells (see (26–29) for reviews). However, it remains generally unknown how cellular machineries select particular rRNA genes to establish their activation or repression.

**Figure 1.**
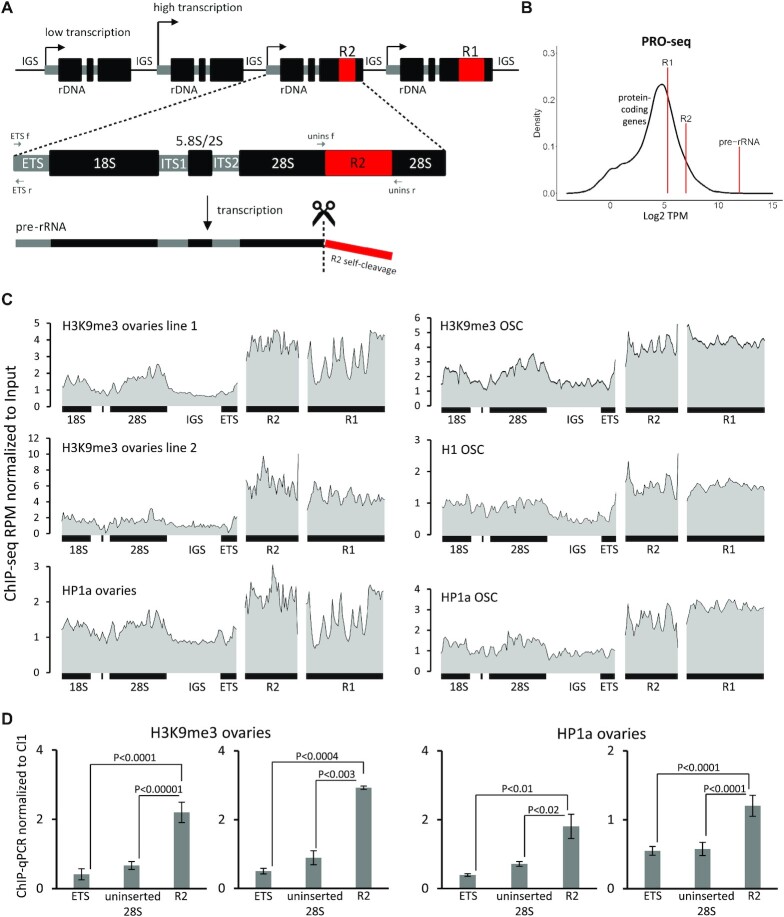
Interrupted rDNA units are enriched in heterochromatin marks compared to intact ones. (**A**) Scheme of the rDNA array fragment in *D. melanogaster*. The rDNA cluster consists usually of 200–250 rDNA units separated by intergenic spacers (IGS). Each rRNA gene is about 8 kb long and encodes 18S, 28S, 5.8S rRNAs and a small 2S molecule. Some rRNA genes contain insertions of R1 and R2 elements in the 28S sequences. R2 retrotransposons are transcribed only as a part of pre-rRNA molecules. A self-cleaving ribozyme releases the 5′-end of the R2 transcript from the upstream 28S rRNA sequence. Locations of primers used for detection of intact (uninserted) rDNA genes (unins f/unins r) and ETS region (ETS f/ETS r) are indicated by arrows. (**B**) Input-normalized levels of transcription of single rDNA repeat, R1 and R2 elements according to ovarian PRO-seq. The distribution of transcription levels (log_2_ TPM) of protein-coding genes is shown in black. (**C**) Density profiles of ChIP-seq reads on rDNA unit, R2 and R1 elements in ovaries and OSC cells. RPMs are normalized to the corresponding input DNA in 100 bp windows. The genotypes of the analyzed *D. melanogaster* lines are indicated in the Materials and Methods. (**D**) ChIP-qPCR analysis of H3K9me3 and HP1a levels in ovaries of two *D. melanogaster* lines. The enrichment levels of uninserted 28S sequence, R2 insertions and the beginning of ETS, which corresponds to promoter regions of both inserted and uninserted rDNA units, are normalized to the heterochromatic cluster-1 (Cl1, 42AB). Mean ± s.d. and *P*-values based on Student’s *t*-test are indicated.

*Drosophila* provides an attractive model for studying the transcriptional regulation at the level of individual rDNA units because some rRNA genes in this organism are marked by insertions of non-LTR retrotransposons called R1 and R2 (see (30,31) for reviews). In *D. melanogaster*, rDNA clusters located in the X and Y chromosomes usually consist of 200–250 rDNA units (32), more than half of which contain insertions. R1 elements occupy from 15% to 67% of rRNA genes, and R2 up to ∼ 30% depending on *D. melanogaster* strain (33). The R2 element is highly conserved being present in the genomes of various groups of animals but not found in mammals (30,34). R2 insert into a strictly defined site in the rDNA sequence located 2651 bp after the beginning of the 28S rRNA and R1 elements are integrated 74 bp downstream of the R2 insertion site (30). R2 elements are of particular interest because they are present exclusively at the rDNA locus, whereas R1 insertions have also been found in other regions of the genome (35–38). Along with unusually high specificity of integration, another peculiarity of R2 elements is that they do not have their own promoters and therefore can be transcribed only as a part of pre-rRNA (39–42). Hence, R2 sequences can be considered as markers of the expression of corresponding rDNA units. Full-length R2 insertions encode a ribozyme, which releases the 5′-end of the R2 transcript from the upstream 28S rRNA sequence during the autocatalytic reaction (Figure 1A), whereas the mechanism of 3′-end formation of R2 RNA remains unclear (30,42). The R2 protein encoded by this element has several domains, including DNA- and RNA-binding motifs, a reverse transcriptase and an endonuclease (30,43,44).

Given that R2 elements hijack the most potent transcription machinery in eukaryotic cells, namely Pol I, they are likely to be under especially strong control from cellular silencing mechanisms. With complete release of transcriptional and post-transcriptional repression, one would expect an accumulation of their transcripts comparable to rRNA abundance. On the other hand, some level of R2 expression may be beneficial for the host, since it has been suggested that the R2 endonuclease can initiate recombination restoring rDNA clusters in cells with decreased rDNA copy number (45,46). In fact, it has been known for several decades that rRNA genes with insertions (hereafter, inserted or interrupted rDNAs) produce hundreds or thousands of times fewer transcripts than intact (uninserted) rDNAs (47–49), though in one work only a 10-fold difference was observed (50). Electron microscopy using the ‘Miller spreading technique’ (6) showed that the low level of R2 transcripts is mainly due to the transcriptional repression of the entire rDNA units, which contain insertions (51,52). However, run-on analysis indicated that transcription is often terminated within the insertion sequence (50). Furthermore, it was found that most uninserted rRNA genes are also silenced: psoralen cross-linking assay revealed that only <10% of the total rDNA units are actively transcribed (50). The mechanism of inserted rDNA recognition and repression remains obscure, though some assumptions have been made in a number of reports (40,53,54). In particular, the repression was supposed to engage small RNAs and local formation of heterochromatin (30), i.e. the pathways that are known to determine silencing of conventional transposable elements (TEs) in the *Drosophila* genome (55–57). An upregulation of R1 and R2 (usually several times relative to controls) was observed in some experimental systems due to the impairment of Ago2-siRNA and Piwi-piRNA pathways (58–61), H1 histone (62), lamin (63), the architectural protein CTCF (53) and the nucleolar protein Nopp140 (64). An analysis of the proportion and interposition of inserted and uninserted rRNA genes within clusters suggested a ‘transcriptional domains’ model according to which the region of the rDNA cluster lacking R1/R2 insertions is selected to be transcriptionally active, while the rest of the locus undergoes heterochromatinization (40,65). However, differences in chromatin density and histone modifications between inserted and uninserted rDNA units have not been observed in embryonic cells (50). Nevertheless, another report found the colocalization of R1 and R2 elements with condensed chromatin in the nucleolus of salivary gland cells (38). Overall, the role of chromatin context in the repression of interrupted rDNA units and in the general regulation of *Drosophila* rDNA transcription remains controversial and under-investigated.

Here, we study the role of the heterochromatic state in the silencing of ribosomal genes with TE insertions in *D. melanogaster* ovarian germ cells that are characterized by an especially intense rRNA synthesis required for the oocyte development (66,67). We found that interrupted rDNA units are indeed enriched with H3K9me3 and HP1a repressive marks compared to intact rDNA repeats. However, disruption of H3K9 methylation or depletion of HP1a led to only a modest upregulation of interrupted rRNA genes, indicating that selective silencing of rDNA units is mainly determined by other mechanisms. We revealed a drastic R2 derepression up to several hundred folds due to the impairment of different components (Udd, TAF1b and TAF1c-like) of the SL1-like complex participating in Pol I transcription initiation (68). H3K9me3 was reduced at derepressed R2-inserted rDNA units in *udd* mutants. On the contrary, inhibition of transcription by actinomycin D caused an increase of the H3K9me3 level in the chromatin of uninserted rRNA genes. Overall, we propose that intact rRNA genes can be selected based on a transcriptional feedback loop, whereas less effectively transcribed rDNA copies undergo heterochromatinization. We also hypothesize that enhanced R2 expression due to the defects of SL1-like can be a part of an adaptive response to reduced rRNA production.

## MATERIALS AND METHODS

### *Drosophila* strains, maintenance and crosses

*Drosophila melanogaster* stocks and crosses were maintained under standard conditions at 25°C or in some cases, as indicated below, at 18°C. *udd^1^* and *udd^null^* lines were kindly provided by M. Buszczak (68). *udd^0683-G4^* stock with genotype *w^1118^; PBac{IT.GAL4}udd^0683-G4^* was obtained from Bloomington Drosophila Stock Center (BDSC #63478). For analysis of *udd* mutant phenotype, *udd^1^/udd^null^* or *udd^0683-G4^*/*udd^null^* flies were compared to heterozygotes (designated as *udd/+*) obtained in the same cross (mix of *udd^1^*/+ and *udd^null^/+* or *udd^0683-G4^* and *udd^null^*, respectively). Because of the increasing with age loss of germ cells in *udd^1^/udd^1^* and *udd^1^/udd^null^* ovaries, for most experiments the ovaries from 0- to 1-day-old females were isolated. To check whether the *udd* mutant alleles exhibit haploinsufficiency, by genetic crosses, we obtained flies with X-chromosomes carrying rDNA clusters from *udd* mutant lines and chromosomes 2 lacking *udd* mutations and compared them with corresponding *udd* heterozygotes. For ChIP-qPCR analysis of H3K9me3 and HP1a marks we used the wild-type *Batumi* strain and *piwi^2^*/+; *piwi^Nt^*/+ flies exhibiting the wild-type ovary phenotype (69). The use of this line was due to the previously obtained H3K9me3 and HP1a ChIP-seq data (70). The wild-type *Canton-S* line was used as a control in RT-PCR experiments.

UAS-RNAi stocks were obtained from BDSC and Vienna Drosophila Resource Center (VDRC): egg RNAi (#101677 VDRC, #109673 VDRC), Su(var)3–9 RNAi (#39378 VDRC, #101494 VDRC), HP1a RNAi (#31994 VDRC, #31995 VDRC), white RNAi (#30033 VDRC), Udd RNAi (#25313 VDRC), TAF1B RNAi (#61957 BDSC, #105873 VDRC), TAF1C-like RNAi (#62424 BDSC). Germline knockdowns (GKD) were induced by crossing these lines with *nos-GAL4* driver *P*{*UAS-Dcr-2.D*}*1*, *w^1118^*, *P*{*GAL4-nos.NGT*}*40* (#25751 BDSC), providing germline-specific GAL4 expression under the control of the *nanos* (*nos*) gene promoter. The resulted RT-PCR values for egg, Su(var)3–9 and HP1a GKD were averaged based on three biological replicates for each of the two RNAi lines. HP1a crosses were grown at 18°C, since maintaining at 25°C resulted in almost complete loss of germ cells and other GKD were grown at 25°C. To obtain Su(var)3–9/egg double GKD, RNAi constructs for both proteins were combined by genetic crosses and the resulting line was crossed with *nos-GAL4* driver. Females of 4 offspring genotypes containing the same rDNA clusters were analyzed: Su(var)3–9 GKD, egg GKD, double GKD and non-KD individuals.

Fly stocks for histone replacement system (71) were kindly provided by Robert J. Duronio. These stocks contain a transgenic histone cluster, in which codons for K9 lysine residues of the H3 histone genes are substituted with arginine (K9R) or a wild-type transgenic histone cluster (HWT). Progeny lacking endogenous histone genes and containing K9R or HWT transgenes integrated on chromosome 3 was produced by crossing parents heterozygous for the *HisC* deletion on chromosome 2 and identified by YFP expression using UAS-GAL4 as described (71). For this, *ΔHisC, twi-GAL4/CyO* females were crossed with *ΔHisC, UAS-2xEYFP/CyO; HWT/HWT* or *K9R/K9R* males. Therefore YFP-positive progeny carried *HisC* deletion on both chromosomes 2. Then YFP-positive HWT and K9R larvae were manually selected and processed for RT-qPCR analysis. The absence of H3K9me3 modification in K9R larvae was confirmed by immunostaining of polytene chromosomes from salivary gland cells with H3K9me3 antibodies (Upstate, 07–442).

### Chromatin immunoprecipitation (ChIP) and ChIP-seq

ChIP was performed according to the published procedure (72) with some modifications. Formaldehyde cross-linking was performed immediately after manual ovary dissection without freezing, that was found to be important to obtain high enrichments. Chromatin was fragmented by sonication on Vibra-Cell (Sonics) with amplitude 15% during 35 pulses of 10 s with 10 s pause intervals. About 0.6 μg of chromatin extract was taken for DNA input and stored at −20°C. For IP, 2 μg of chromatin extract was diluted up to 500 μl in lysis buffer and pre-incubated overnight at 4°C in the presence of 50 μl Protein A-Sepharose suspension (Amersham Pharmacia, 50% w/v, hereinafter PAS). After removing PAS, samples were incubated at 4°C for 5 h with antibodies and overnight with the addition of PAS. Subsequent steps were performed as described (72). The following antibodies were used: rabbit anti-H3K9me3 (Upstate, 07–442), rabbit anti-HP1a (a gift from Sarah Elgin), rabbit anti-H4K20me3 (Abcam, ab9053), rabbit anti-H3K27me3 (Upstate, 07–449), guinea pig anti-Udd (a gift from Michael Buszczak (68)). The obtained qPCR values were normalized to those of the DNA input and the region of cluster 1 (Cl1)/42AB as a control genomic region using the following formula: V(target)IP * V(Cl1)Input / V(Cl1)IP * V(target)Input, where V is qPCR value. For each value, 3 to 7 biological replicates were analyzed. PCR primers are shown in Supplementary Table S1.

ChIP-seq libraries were prepared from samples of precipitated DNA using Accel-NGS 1S Plus DNA Library Kit (Swift Biosciences) and sequenced on the Illumina HiSeq 4000 platform on the basis of the Genomics Core Facility of Skolkovo Institute of Science and Technology (Moscow). ChIP-seq data were deposited in the NCBI Gene Expression Omnibus (GEO) under the accession number GSE183035.

### qPCR and RT-qPCR

Total RNA was extracted from hand-dissected ovaries or other organs using TRIzol reagent (Invitrogen). DNase treatment and reverse transcription was performed as described (61). Genomic DNA was isolated from the whole flies according to the standard procedure (73). At least three biological replicates of RNA and genomic DNA were used. Nucleic acid quantification was performed using NanoDrop 1000 spectrophotometer V3.8 or using the Qubit RNA BR Assay Kit (Invitrogen). Both genomic DNA and cDNA were analyzed in triplicates on DT96 real-time DNA amplifier (DNA-Technology) using SYTO™ 13 Green Fluorescent Nucleic Acid Stain (Invitrogen) and Hot Start Taq DNA polymerase (Evrogen) applying a program of 94°C, 5 min, followed by 40 cycles at 94°C, 15 s; 60–64°C (depending on primers), 20 s; 72°C, 20 s, except for primers to truncated insertions of R1 and IGS-ETS cotranscripts, for which the last step was 72°C, 60 s. A melting curve analysis was performed from 72 to 92°C, with 0.3°C increments. Relative expression was calculated from *C*q values using a ΔΔ*C*q method. RT-qPCR values were normalized to the rp49 mRNA level, which according to our observations was not significantly changed in a number of RNA-seq data sets, including those for HP1a GKD and *udd* mutant ovaries. Actin 5C (Act5C) mRNA was used as an additional control for RT-qPCR. To compare transcript levels between different regions of the R2 element, we calculated ratios of the rp49-normalized RT-qPCR values to genomic qPCR values normalized to the rp49 gene for the same *Drosophila* genotype. PCR-products were verified by gel electrophoresis and in some cases by Sanger sequencing. For the design of primers corresponding to rDNA locus and inserted TEs we used the following sequences: rDNA (clone pDm238, NCBI M21017.1), R2 (X51967.1) and R1 (X51968.1). Sequences of PCR primers, amplification efficiencies, annealing temperatures and amplicon lengths are shown in Supplementary Table S1.

### RNA-seq

Total RNA samples were isolated from ovaries using TRIzol reagent (Invitrogen) according to the standard protocol and treated with DNase using the DNA-free DNA Removal Kit (Invitrogen). rRNA was removed by hybridization of 200 ng of total RNA with biotinylated oligonucleotides complementary to different regions of rRNA followed by binding to Dynabeads™ MyOne™ Streptavidin C1 (Invitrogen). After precipitation, supernatants containing rRNA-depleted RNA were precipitated with isopropanol. NGS libraries were prepared in duplicates using MGIEasy RNA Library Prep Set V3.1 (MGI Tech Co. Ltd.) according to the manufacturer’s recommendations. Sequencing was carried out on the basis of the Center for Precision Genome Editing and Genetic Technologies for Biomedicine of the Pirogov Russian National Research Medical University (Moscow) with 2 × 100 bp paired-end reads on a MGISEQ-2000 platform (MGI Tech Co. Ltd.). RNA-seq data were deposited in the NCBI GEO under the accession number GSE183035.

### Bioinformatic analysis

The following ChIP-seq data and corresponding input DNA samples were analyzed: H3K9me3 and HP1a ChIP-seq from *piwi^2^*/+;*piwi^Nt^*/+ ovaries #GSE56347 (70) (designated as line 1); H3K9me3 ChIP-seq from shWhite ovaries #GSE43829 (74) (designated as line 2); H3K9me3, HP1a and H1 ChIP-seq from Control-KD OSC #GSE81434 (75), Udd ChIP-seq from *udd^0683-G4^/udd^null^* and *udd/+* ovaries performed in this work. A quality control of reads and trimming of adapters for ChIP-seq and PRO-seq/RNA-seq data were carried out using FastQC version 0.11.5 and Trimmomatic version 0.39 (http://usadellab.org/cms/?page=trimmomatic/), respectively. Read mapping to the *D. melanogaster* reference genome (release 5.57 from flybase.org), rDNA (NCBI M21017.1), R1 (X51968.1), and R2 (X51967.1) sequences was performed using Bowtie2 version 4.1 (http://bowtie-bio.sourceforge.net/index.shtml/) with the mapping option -k1. Further filtering and indexing were carried out using Samtools version 1.7 (http://www.htslib.org/). To create coverage profiles, the data were converted to reads per million (RPM) and the region of interest was divided into 100 nt windows. Further normalization of ChIP-seq and PRO-seq to input was performed using bedtools version 2.26.0 (https://bedtools.readthedocs.io/en/latest/). PRO-seq data for *w^1118^* (wild-type) ovaries (76) was taken from GEO #GSM3608097 and normalized to DNA (ChIP-seq input sample from *w^1118^* ovaries #GSM3608100). To obtain transcript per million (TPM) for PRO-seq and RNA-seq data, Salmon version 1.3.0 (https://salmon.readthedocs.io/en/latest/salmon.html/) (77) was used with the size of the kmer = 31. The data were visualized using the ggplot2 package (https://ggplot2.tidyverse.org/).

### Cell line and actinomycin D treatment

Ovarian somatic cells (OSC) were grown at 25°C in Shields and Sang M3 insect medium (Sigma-Aldrich **#**S3652) supplemented with 10% heat inactivated fetal bovine serum (FBS) (Gibco #10270106), 10% fly extract, 10 μg/ml insulin (Sigma-Aldrich # I9278), 0.6 mg/ml L-glutathione (Sigma-Aldrich #G6013), 50 units/ml penicillin and 50 μg/ml streptomycin. To prepare fly extract, 1 g of flies, 3–7 days after eclosion, were homogenized in 6.8 ml of M3 medium and centrifuged for 15 min at 1500 *g*. The supernatant was heated for 10 min at 60°C, centrifuged and the pellet was discarded. Actinomycin D (ActD) (Sigma #A9415) was used in the final concentration of 10 μg/ml in OSC growth medium for 2 h at 25°C. ActD at lower concentration (1 μg/ml) did not lead to complete block of nascent transcription in the nucleolus.

### RNA FISH, immunostaining, EU and EdU incorporation assays, TUNEL assay

RNA FISH with an R2 probe was performed using tyramide signal amplification as previously described (78). The DNA template for R2 probe transcription carrying the T7 RNA polymerase promoter sequence at their 5′-end was PCR-amplified on the *Drosophila* genomic DNA using primers indicated in Supplementary Table S1. Single molecule RNA FISH (smFISH) for R2 and pre-rRNA was carried out using Cy3- and CY5-labeled oligonucleotide probes, respectively (Supplementary Table S1). Dissected ovaries were fixed in 4% PFA, then washed in PBS with 0.1% Tween20 and 0.3% Triton X100. Ovaries were incubated in buffer F containing 30% formamide, 2× SSC and 0.1% Tween20, briefly heated (80°C) in 100 μl of hybridization buffer containing 30% formamide, 2× SSC, 0.1% Tween20, 10 μg herring sperm DNA, 10 μg yeast tRNA, 0.1 mM DTT and 15 ng DNA labeled probes and then incubated overnight at 37°C. Ovaries were washed in buffer F with decreasing concentration of formamide (30%, 15%, 5%) and with PBS.

Immunostaining was performed according to previously described protocols for OSC cells (79) and ovaries (80). The following primary antibodies were used: rabbit polyclonal anti-fibrillarin (1:500, Abcam #5821); mouse monoclonal anti-fibrillarin (1:500, Abcam #4566); rabbit anti-lamin (1:500, provided by P. Fisher) (81), guinea pig polyclonal anti-Udd (1:800, provided by M. Buszczak) (68), rabbit anti-HP1a (a gift from Sarah Elgin), rat anti-Vasa (1:100; DSHB #AB_760351). Secondary antibodies (Invitrogen, Thermo Fisher Scientific) were the following: anti-rabbit IgG Alexa Fluor 488; anti-mouse IgG Alexa Fluor 488; anti-rat IgG Alexa Fluor 488; anti-rabbit IgG Alexa Fluor 546; anti-mouse IgG Alexa Fluor 546; anti-rabbit IgG Alexa Fluor 633; anti-mouse IgG Alexa Fluor 633 and anti-guinea pig IgG Alexa Fluor 633.

The newly synthesized RNA in OSC cells was labeled with 5-ethynyl-uridine (EU) (Life Technologies, #E10345) as described (61). For EU incorporation assay in ovaries, manually dissected ovaries were incubated in the OSC growth medium with 1 mM EU for 1 h at 25°C, then fixed and permeabilized as described (79). EU detection using the Click-iT™ reaction was carried out in a cocktail containing Alexa Fluor 647 azide, triethylammonium salt (#A10277, Invitrogen, Thermo Fisher Scientific) and Reaction Buffer Kit (#C10269, Invitrogen, Thermo Fisher Scientific) for 30 min in the dark at room temperature. Then ovaries were washed in PBTX and processed for immunostaining.

For the 5-Ethynyl-2′-deoxyuridine (EdU) labeling, control OSC cells and cells treated by 10 μg/ml ActD during 2 h were supplied with EdU (10 μM in final concentration) and incubated 1 h at 25°C. The cells were fixed, as described above, and processed for EdU label detection using the Click-iT™ reaction according to the manufacturer’s instructions. Then cells were washed in PBTX and processed for confocal microscopy.

TUNEL staining was performed using Click-iT™ Plus TUNEL Assay for In Situ Apoptosis Detection, Alexa Fluor™ 647 dye kit (#C10619, Invitrigen, Thermo Fisher Scientific) according to the manufacturer’s instructions.

Confocal microscopy was performed using an LSM 510 META system (Zeiss) and LSM 900 Confocal with Airyscan 2 super-resolution detector (Zeiss).

## RESULTS

### R1 and R2 elements are enriched with repressive chromatin marks compared to intact rRNA genes

To clarify the extent to which the interrupted rRNA genes are silenced in ovaries, we evaluated levels of pre-rRNA, as well as R1 and R2 nascent transcripts in publicly available PRO-seq data for the ovaries of the *w^1118^ D. melanogaster* line (76). To account for the copy number of these repetitive elements in the genome, we normalized PRO-seq values to the number of corresponding sequences in the genomic DNA sample of the same *Drosophila* strain (76). We calculated that the *w^1118^* female genome contains a total of 206 rDNA genes, of which 20 (∼10%) have R2 insertions. As noted above, R1 elements can insert outside the rDNA locus and therefore the R2 sequence is more appropriate as a marker of inserted rDNA. Our analysis showed that the average pre-rRNA transcription level per rDNA repeat is higher than transcription of any protein-coding gene, and approximately 100 and 30 times higher than that of R1 or R2 elements, respectively (Figure 1B). In fact, expression levels of R1/R2-inserted and vigorously transcribed intact rRNA genes can differ much stronger, given that the latter constitute only a small fraction of all rDNA units.

Next, we asked whether interrupted rDNA repeats are associated with repressive chromatin marks. To check this, we first analyzed our own (70) and publicly available ChIP-seq data from ovaries (74) and ovarian somatic cultured cells (OSC) (75) using normalization to the DNA input samples of corresponding genomes. We observed a pronounced enrichment of the H3K9me3 heterochromatin mark and a main reader of this mark, HP1a (82), in R1 and R2-associated chromatin compared to the rDNA sequence in all analyzed ChIP-seq libraries (Figure 1C). In addition, R1 and R2 elements exhibited an increased level of H1 linker histone (Figure 1C), which is also mainly associated with repressive chromatin in *Drosophila* (62,75,83). We then performed H3K9me3 and HP1a ChIP-qPCR taking advantage of PCR to detect uninserted rRNA genes using primers surrounding the R1/R2 insertion sites in the 28S rDNA sequence (primers location is shown in Figure 1A). We found a >3-fold higher H3K9me3 level at R2 insertions compared to uninserted rDNA units in the ovaries of two tested *Drosophila* lines and about a 2.5-fold higher enrichment of HP1a (Figure 1D). Interestingly, we also observed reduced H3K9me3 occupancies in the very beginning of ETS (Figure 1D, primer location on Figure 1A), which corresponds to promoter regions of all rDNA units (both inserted and uninserted). This result indicates that chromatin marks are non-uniformly distributed along a single rDNA repeat and promoter regions of rDNA genes are depleted in heterochromatin marks.

### Disruption of heterochromatin only partially weakens the repression of interrupted rRNA genes

**Figure 2.**
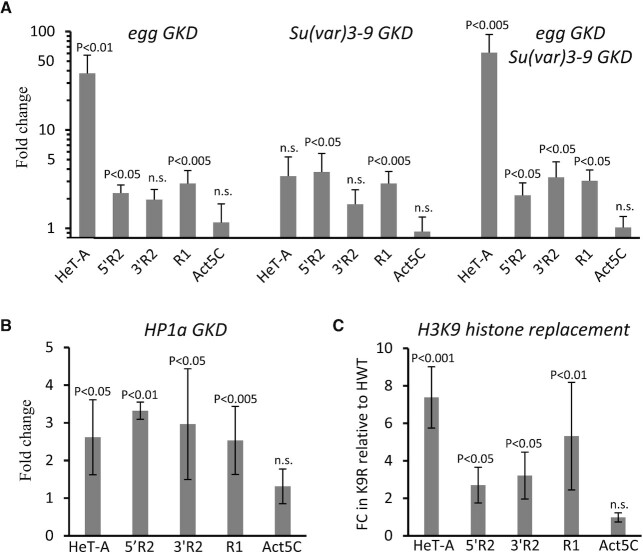
Lack of heterochromatin components exerts a moderate effect on expression of inserted rRNA genes. (**A**) RT-qPCR analysis of HeT-A, R1 and two regions (5′ and 3′) of R2 elements in ovaries upon nos-GAL4 driven germline knockdown (GKD) of Egg, Su(var)3–9 and Egg/Su(var)3–9 double GKD. Mean fold changes (FC) ± s.d. relative to the control non-KD sisters normalized on the rp49 mRNA levels are indicated. Actin5C (Act5C) is shown as an additional control gene. (**B**) RT-qPCR analysis of HP1a GKD ovaries. (**C**) RT-qPCR analysis of larvae lacking H3K9 methylation. Mean FC ± s.d. of RT-qPCR values for larvae with H3K9 replacement (K9R) relative to control individuals containing a transgenic wild-type histone array (HWT) is shown. RNA levels were normalized to rp49 mRNA. *P*-values are based on Student’s *t*-test, n.s. = not significant.

To estimate the impact of repressive chromatin state on the silencing of rDNA units with TE insertions, we analyzed the levels of R1 and R2 transcripts in ovaries depleted for H3K9-specific methyltransferases (HMTs) and HP1a. According to RT-qPCR, nos-GAL4 driven germline knockdown (GKD) of H3K9 methyltransferase Eggless/SetDB1 (Egg) led to about a 2- to 3-fold upregulation of R1 and R2 elements as compared to the non-KD sisters (Figure 2A). Note that Egg was previously shown to be strongly required for transcriptional silencing of a broad range of TEs in ovaries (84) and its knockdown caused about a 40-fold derepression of telomeric HeT-A element in our analysis (Figure 2A). GKD of another HMT, Su(var)3–9 known to be involved in the formation of constitutive heterochromatin and the spreading of the H3K9me3 mark (84–86) led to much more severe defects of oogenesis than Egg depletion but also induced a 2–3-fold upregulation of R1 and R2 elements (Figure 2A). Double GKD of Egg and Su(var)3–9 led to activation of R1 and R2 at the same level as single knockdowns (Figure 2A), indicating that two HMTs do not substitute for each other in regulation of rRNA genes. Similarly, the levels of R1 and R2 transcripts increased approximately 3-fold upon HP1a GKD (Figure 2B and Supplementary Figure S1). Then, to directly evaluate the role of H3K9 methylation in the repression of inserted rDNA units we took advantage of a histone replacement system (71) where a wild-type cluster of histones is replaced by an artificial one, in which codons for K9 lysine residues of the H3 histone genes are replaced with arginine (K9R) (see Materials and Methods for details). Although this replacement abolishes all modifications of H3K9, it mainly manifests itself in derepression of TEs likely due to the loss of H3K9me2/3 marks (71). Homozygous K9R individuals survive up to the larval stage. RT-qPCR analysis of female larvae showed that K9R substitution induced an approximately 3-fold increase of R2 expression, respectively, as compared to control individuals (HWT) containing a wild-type histone transgenic array (Figure 2C). Thus, derepression of R1 and R2 upon loss of heterochromatin components is much less than the upregulation that would be expected if the silencing of inserted rRNA genes was completely disrupted. This revealed that the putative mechanisms of individual rDNA unit repression mostly retain their effectiveness in the absence of H3K9me3/HP1a heterochromatin marks.

### Impairment of components of the SL1-like complex leads to drastic upregulation of R2 elements

**Figure 3.**
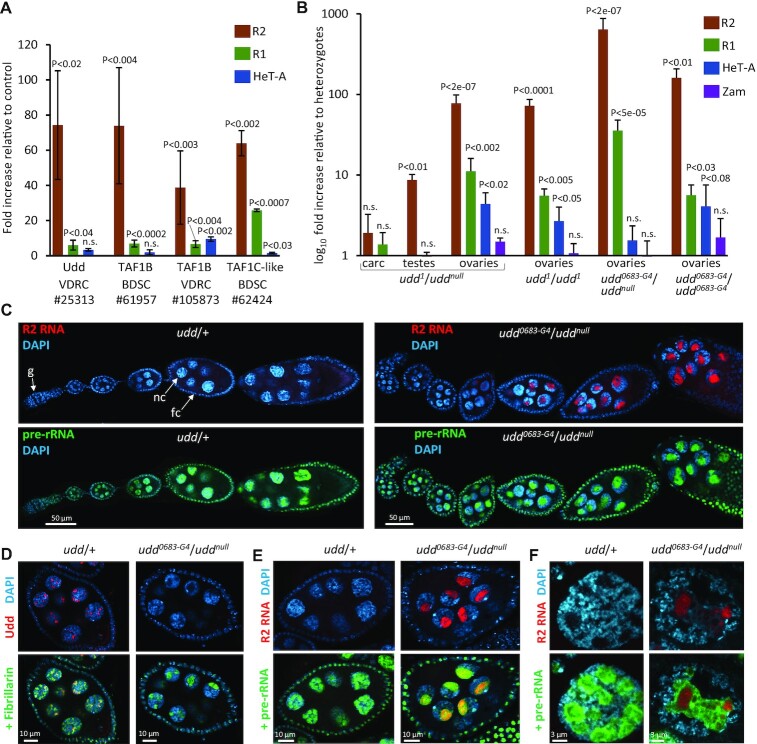
Impairment of SL1-like components cause derepression of R1 and R2 elements in ovaries. (**A**) RT-qPCR analysis of R2 3′-region, R1 and HeT-A elements in ovaries upon nos-GAL4 driven GKD of SL1-like subunits. ID numbers of UAS-RNAi stocks obtained from BDSC or VDRC resource centers are indicated. RNA levels were normalized to rp49 mRNA and fold increases were calculated relative to the corresponding control sisters. *P*-values from Student’s *t*-test for differences between GKD and controls are shown, n.s. = not significant. (**B**) RT-qPCR analysis of R1 and R2 elements in carcasses (carc), testes, and ovaries of *udd* mutants. HeT-A and Zam were analyzed as examples of TEs expressed in the germline and somatic ovarian cells, respectively. Mean fold increase ± s.d. relative to the corresponding heterozygous sisters normalized on the rp49 transcript levels are indicated. *P*-values from Student’s *t*-test for differences between homo- or transheterozygotes and heterozygotes are shown. (**C**) smFISH of R2 transcripts and pre-rRNA in ovarioles of *udd^0683-G4^*/*udd^null^* and control heterozygotes (*udd*/+). A germarium region is indicated as ‘g’; examples of germline nurse cells and somatic follicle cells are indicated as ‘nc’ and ‘fc’, respectively. (**D**) Immunostaining of stage 7 egg chambers for nucleolar marker fibrillarin and Udd. Nucleoli appeared as dark holes in DAPI stained nuclei. (**E**) smFISH of R2 transcripts and pre-rRNA in stage 6 egg chambers. (**F**) Super-resolution image of nurse cell nuclei of stage 9–10 egg chambers showing distribution of R2 and pre-rRNA transcripts detected by smFISH.

We then examined a role of the Pol I transcription initiation apparatus in the regulation of inserted rDNA units. Transcription of rRNA genes in mammals requires several specific basal factors: upstream binding factor (UBF), the RRN3 (Tif-IA) protein and the core pre-initiation complex SL1, consisting of the TATA-box binding factor (TBP) and TBP-associated factors (TAFs). SL1 specifically recognizes and binds rDNA promoter sequences (87). *Drosophila melanogaster* lacks UBF homolog, while Tif-IA and the SL1-like complex were described in the fruit fly (68,88). Three components of the *Drosophila* SL1-like complex were identified: conserved TAF1B and TAF1C-like subunits and a small 18 kD protein called Udd (Under-developed) (68), which does not contain any known motifs and has no homologs outside the *Diptera*. It has been shown that the disruption of SL1-like compromised rRNA synthesis and development of ovarian germ cells (68). Although the SL1-like complex is thought to be pivotal for the rDNA transcription, which mediates the expression of TEs incorporated into pre-rRNA, we surprisingly found that germline knockdowns of TAF1B, TAF1C-like and Udd led to 40- to 70-fold upregulation of R2 and to a lesser extent of R1 elements (up to 26-fold) (Figure 3A). RT-qPCR also unexpectedly revealed that total levels of pre-rRNA (18S-ITS1 cotranscripts) were not changed or fluctuated slightly (no >1.5-fold) in ovaries depleted for SL1-like components (Supplementary Figure S2). Note that pre-rRNA can be derived from both inserted and uninserted rDNA units. Thus, given the increased contribution of inserted rDNA unit transcription to the total pre-rRNA pool, intact rRNA genes may be downregulated upon knockdowns. These results suggest that impairment of the SL1-like complex affects the selection of individual rDNA units for activation or silencing.

We focused on investigating the effects of Udd lack due to the existence of viable *udd* mutants. We examined the previously described *udd^1^* hypomorphic allele (68), and the unstudied before *udd^0683-G4^* allele (BDSC #63478) combining them with the *udd^null^* allele (68). It is noteworthy that homozygous *udd^null^/udd^null^* mutation with complete loss of Udd is lethal (68). All other tested *udd* mutant flies (*udd^1^/udd^1^*, *udd^1^/udd^null^*, *udd^0683-G4^*/*udd^null^* and *udd^0683-G4^*/ *udd^0683-G4^*) were sterile and had reduced ovaries and testes (in accordance with (68)) but did not display any morphological body defects known to be associated with significantly decreased ribosome production (minute-like phenotypes (89)). RT-qPCR revealed a 70- to 80-fold upregulation of R2 elements in *udd^1^/udd^1^* and *udd^1^/udd^null^* ovaries, relative to corresponding heterozygotes (Figure 3B). Even stronger derepression of R2 (>500-fold) was observed in *udd^0683-G4^*/*udd^null^* ovaries (Figure 3B). The amount of R1 transcripts was increased from 5- to 36-fold in ovaries of various *udd* mutant combinations. Expression of other analyzed TEs showed a smaller increase or no change (Figure 3B). No substantial upregulation of R1 and R2 elements was observed in *udd*/+ ovaries compared to ovaries of wild-type flies carrying the same rDNA clusters on X-chromosomes (Supplementary Figure S3). Although Udd is expressed ubiquitously and immunostaining shows Udd within nucleoli in various *Drosophila* tissues (Supplementary Figure S4), we did not found any significant influence of *udd* mutations on R2 expression in carcasses (bodies without gonads), while about a 10-fold increase of R2 RNA level was revealed in *udd^1^/udd^null^* testes (Figure 3B).

We examined in which types of ovarian cells R2 activation occurs in *udd* mutants. *Drosophila* ovaries are composed of ovarioles, chains of egg chambers starting from a germarium region and then consistently developing over 14 stages. Each egg chamber includes 16 cytoplasmically connected germline cells (fifteen nurse cells and a single oocyte) surrounded by somatic follicle cells. Nurse cells are polyploid (up to 8000C) and exhibit high transcription activity supplying the transcriptionally inert oocyte with proteins, RNA and ribosomes through intercellular channels (90). Consistent with previous report (68), we noted that ovaries of *udd^1^/udd^1^* and *udd^1^/udd^null^* flies contain egg chambers only up to stages 4–5 of oogenesis. *udd^0683-G4^*/*udd^null^* ovaries had a more normal phenotype and retained oogenesis up to stage 11 (Figure 3С and Supplementary Figure S5). Egg chambers of later stages were lost likely as a result of germ cell apoptosis (Supplementary Figure S5) and consequently *udd^0683-G4^*/*udd^null^* females did not produce embryos. RNA FISH revealed the accumulation of R2 transcripts mainly in the nucleoli of nurse cells in both *udd^0683-G4^*/*udd^null^* (Figure 3С) and *udd^1^/udd^null^* ovaries (Supplementary Figure S6A), whereas weak R2 RNA signals were also observed in some follicle cell nuclei (Supplementary Figure S6B). In the control heterozygotes, R2 RNA was not detected by FISH in any cells. Remarkably, in both *udd^0683-G4^*/*udd^null^* and *udd^1^/udd^null^* ovaries FISH signals were faint in germ cells of germarium and early egg chamber stages, then gradually increased as oogenesis progressed and reached a maximum in nurse cells at the latest stages present in mutants (Figure 3С and Supplementary Figure S6A). Therefore, a much stronger R2 upregulation in *udd^0683-G4^*/*udd^null^* compared to *udd^1^/udd^null^* ovaries observed by RT-qPCR was likely due to the passing the late oogenesis stages. It is noteworthy that nucleoli in nurse cells develop into branched structures that are different from the ordinary spherical nucleoli of diploid cells (67). However, the nucleoli in nurse cells of *udd* mutants were less branched and appeared as large intranuclear bodies (Figure 3D–F). Interestingly, R2 transcripts tended to accumulate in the central part of the nucleolus, while pre-rRNA was localized mainly at its periphery (Figure 3F).

**Figure 4.**
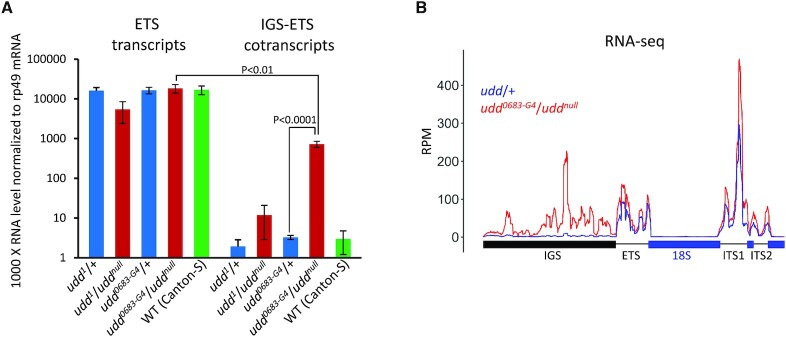
*udd* mutations do not abolish rRNA transcription start site selection but leads to accumulation of intergenic spacer transcripts (IGS RNA). (**A**) RT-qPCR quantification of transcripts corresponding to the beginning of ETS region and IGS-ETS cotranscripts in *udd^0683-G4^*/*udd^null^,udd^1^*^/^*udd^null^* and control *udd*/+ ovaries. Mean ± s.d. and *P*-values based on Student’s *t*-test are indicated. (**B**) RNA-seq profile of the rDNA unit in *udd^0683-G4^*/*udd^null^* and control *udd*/+ ovaries. Reads corresponding to mature rRNAs (18S, 5.8S, 2S, 28S) are removed.

According to RNA FISH and visualization of nascent RNA by EU incorporation assay, nucleolar transcription was observed in germ cells at all oogenesis stages, which are present in the *udd* mutants (Figure 3С and Supplementary Figure S7A). Although a severe decline in rRNA synthesis was previously described for the *udd^1^*/*udd^null^* mutant (68), our RT-qPCR analysis showed about a 2-fold reduction of the ETS and 18S-ITS1 transcripts in *udd^1^*/*udd^null^* ovaries (Figure 4A and Supplementary Figure S7B). No significant alteration of these transcripts was revealed in *udd^0683-G4^*/*udd^null^* ovaries relative to heterozygotes (Figure 4A and Supplementary Figure S7B). However, as noted above, the unchanged level of total pre-rRNA upon the activation of the R1 and R2 elements does not exclude that transcription of intact rDNA units is reduced. Given that the SL1 complex is known to recognise the rDNA core promoter and enable rRNA transcription start at the 5′ end of the ETS (87), we examined whether rRNA transcription in *udd* mutants could be aberrant. We observed an increase of chimeric IGS-ETS cotranscripts in *udd^1^/udd^null^* and notably in *udd^0683-G4^*/*udd^null^* ovaries by RT-qPCR (Figure 4A). Nevertheless, the amount of IGS-ETS RNA was still about 25-fold lower than the amount of transcripts corresponding to the beginning of the ETS in the *udd^0683-G4^*/*udd^null^* ovaries, whereas for the *udd^1^/udd^null^* mutant this difference was greater (Figure 4A). We also performed RNA-seq, which revealed highly elevated level of IGS-derived RNA in *udd^0683-G4^*/*udd^null^* ovaries but also demonstrated that transcription from the beginning of the ETS occurs in the mutant and control ovaries at the same level (Figure 4B). Thus, analyzed *udd* mutants mostly retain normal rRNA transcription start site, while display elevated IGS RNA synthesis and strong upregulation of R1 and notably R2 elements.

### *udd* mutations cause transcriptional activation of entire rDNA units containing TE insertions

**Figure 5.**
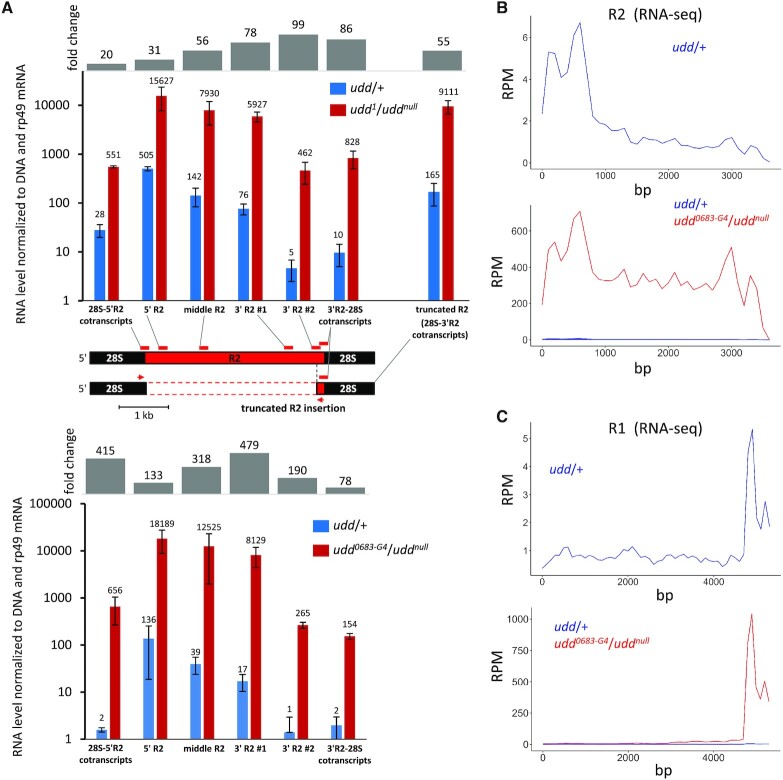
*udd* mutations cause transcriptional activation of entire rDNA units containing TE insertions. (**A**) Fold change and log_10_ RNA level normalized to genomic DNA and rp49 mRNA measured by RT-qPCR in *udd^1^*/*udd^null^* (upper panel) and *udd^0683-G4^*/*udd^null^* (bottom panel) ovaries compared to corresponding heterozygous sisters (*udd*/+). Locations of analyzed PCR fragments on the sequence of the 28S gene containing R2 insertion is indicated by red rectangles. Location of primers used for detection of transcripts corresponding to rRNA genes with truncated R2 insertions is shown by red arrows. Note that 3′ R2-28S cotranscripts correspond to both full-length and truncated R2 elements. Since 3′-ends of the truncated R2 insertions are transcribed more efficiently than in full-length elements, the level of 3′ R2-28S cotranscripts is higher than that of 3′ R2 #2 region, which does not correspond to the truncated R2 copies. (**B**) RNA-seq profiles of the R2 element in *udd*/+ (upper panel) and in both *udd^0683-G4^*/*udd^null^* and *udd*/+ ovaries (bottom panel). The profile does not account for the presence of R2 insertions of different length and therefore the level of transcription at the 3′-end can be overestimated due to the higher abundance of these regions in the genome. (**C**) RNA-seq profiles of the R1 element.

It has been previously shown that the repression of inserted rDNA repeats can occur through termination of transcription within the insertion sequence (50) or due to the transcriptional silencing of the entire rDNA unit (51). To test which of these repression modes may be affected by *udd* mutations, we measured the amounts of transcripts corresponding to different regions of R2 insertions in the *udd* mutant ovaries and control heterozygotes. Then we normalized the RT-qPCR values to qPCR values obtained using the same primers on the genomic DNA of the same *Drosophila* line. Hence, the results shown in Figure 5A allow us to compare the transcript levels between different regions of R2 regardless of their abundance in the genome and primer amplification efficiency. The normalized RT-qPCR values were drastically higher in the *udd^1^*/*udd^null^* and *udd^0683-G4^*/*udd^null^* ovaries than in the controls for all analyzed regions indicating increased transcription along the entire length of R2 insertions (Figure 5A). The transcripts containing the junction of the upstream 28S rRNA and the beginning of the R2 sequence (hereafter, ‘28S-5′R2 cotranscripts’) were also upregulated in both analyzed mutants. Notably, an approximately 400-fold increase of 28S-5′R2 cotranscripts was observed in *udd^0683-G4^*/*udd^null^* ovaries (Figure 5A). Thus, derepression occurs mostly as a result of the enhancement of rDNA transcription upstream of the insertion region. Note that the absolute level of 28S-5′R2 cotranscripts is lower than that of 5′ R2 body transcripts (Figure 5A) because R2 RNA can self-cleave from the 28S rRNA sequence. At the same time, we observed a reduction of the transcript amount in the 5′- to 3′ direction of the R2 body by both RT-qPCR (Figure 5A) and RNA-seq (Figure 5B). In *udd*/+ ovaries, the amount of transcripts detected in the 5′-region of the R2 was approximately 100-fold higher than at its very 3′-end according to RT-qPCR. This difference was only slightly smoothed out in *udd* mutants (Figure 5A). Thus, the lack of Udd strongly enhances transcription across R2-inserted rDNA unit but does not prevent transcriptional decline within the R2 body. Altogether, these results suggest that normally repression of R2-inserted rRNA genes occurs both by silencing of the entire rDNA unit and by interruption of transcription in the R2 body, while *udd* mutations release the repression only on the level of the entire rDNA repeat possibly affecting the transcription initiation step.

Some rDNA units contain R1/R2 insertions shortened to varying degrees from the 5′-end, formed due to abortive reverse transcription (30,41). Using genomic DNA PCR with subsequent sequencing, we detected the highly truncated R2 insertions of ∼180 and ∼50 bp in the *udd^1^*/*udd^null^* genome, whereas full-length R2 is about 3.6 kb. RT-qPCR using a forward primer located in upstream 28S rRNA sequence and reverse primer in the 3′-end of R2 demonstrated that rDNA units with these truncated R2 inserts were derepressed in *udd^1^*/*udd^null^* ovaries similarly to ones with the full-length R2 elements (Figure 5A). Thus, effect of Udd on R2-inserted rDNA units is independent of the insertion length and therefore is unlikely to be determined by any specific nucleotide motifs within the R2 sequence, or at least within its most part. In addition, this result further supports the conclusion that *udd* mutations cause transcriptional activation of entire rDNA units with insertions.

As noted above, the lack of Udd and other SL1-like subunits led to considerably weaker upregulation of R1 than R2 elements according to RT-qPCR (Figure 3A and B). However, RNA-seq showed that a short region corresponding to the 3′-UTR of the R1 element was 200–300-fold derepressed in *udd^0683-G4^*/*udd^null^* ovaries, whereas the level of transcripts derived from the most of this element increased much less drastically (Figure 5C, bottom panel). In *udd*/+ ovaries, the 3′-UTR region was also expressed higher than the rest of the element (Figure 5C, upper panel). We assumed that this effect can be attributed to short insertions of the R1 solo 3′-UTR lacking the R1 body. Indeed, we detected rDNA units containing the 3′-UTR of the R1 element by PCR of *udd^0683-G4^*/*udd^null^* genomic DNA and demonstrated by RT-qPCR that these units were 200-fold upregulated in *udd^0683-G4^*/*udd^null^* ovaries (Supplementary Figure S8). Note that RT-qPCR in Figure 3A,B detected transcription of the R1 middle part. Thus, the truncated R1 insertions are much more strongly derepressed in *udd* mutants when compared to full-length R1 insertions suggesting that the R1 sequence upstream of the 3′-UTR may contain a putative silencer element, which prevents the complete activation of full-length R1 elements in *udd* mutants. This may also be due to the Pol I pausing signal, the presence of which at the R1 5′-end was recently proposed (54).

### Udd is associated with intact but not interrupted rDNA units

**Figure 6.**
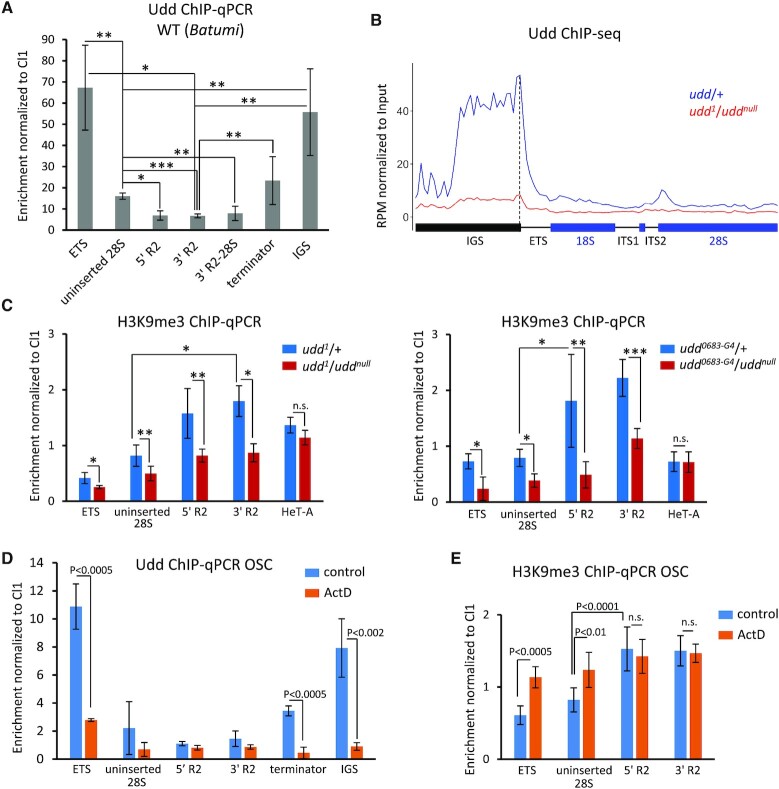
Udd indirectly affects heterochromatinization of rDNA units. (**A**) ChIP-qPCR analysis of *Batumi* wild-type ovaries showing Udd enrichment levels at different regions of rDNA repeats: beginning of ETS; uninserted 28S sequence; 5′ and 3′ regions of R2 insertions; the border of R2 3′-end and 28S sequences (3′ R2-28S); the border of 28S and IGS sequences (terminator); 330bp IGS repeat (IGS). Mean ± s.d. and *P*-values based on Student’s *t*-test are indicated, * *P* < 0.003, ** *P* < 0.01, *** *P* < 0.0005, n.s. = not significant. (**B**) Udd ChIP-seq profile over rDNA unit in *udd*/+ and *udd*^*1*^/*udd^null^* ovaries. RPMs are normalized to the corresponding input DNA in 100 bp windows. (**С**) Upper panel: ChIP-qPCR analysis of H3K9me3 level in *udd^1^*^/^*udd^null^* and *udd*/+ ovaries. Bottom panel: the same for *udd^0683-G4^*/*udd^null^*ovaries. Mean ± s.d. and *P*-values are shown as in (A). (**D**) ChIP-qPCR analysis of Udd level in control and actinomycin D (ActD)-treated (10 μg/ml for 2 h) OSC cells. *P*-values are calculated using Student’s *t*-test. (**E**) ChIP-qPCR analysis of H3K9me3 mark in control and ActD-treated OSC cells. *P*-values are shown as in (D), n.s. = not significant.

Based on our results, it is possible that Udd and other subunits of SL1-like can serve both as transcription initiation factors and components of a hypothetical repressor complex interacted with silent rDNA units. Consistent with the previous report (68), ChIP-qPCR and ChIP-seq showed high Udd enrichment at the rDNA promoter region (ETS on Figure 6A) with the most pronounced peak at the border of IGS and ETS (dotted line in Figure 6B). Udd enrichments at IGS and ETS can correspond to promoters of both inserted and uninserted rDNA repeats. However, by ChIP-qPCR we also observed a weak but significant association of Udd with uninserted 28S rRNA sequences that was about 2-fold higher compared to different parts of R2 insertions in several *D. melanogaster* lines (Figure 6A and Supplementary Figure S9). This effect can be attributed to the preferential interaction of Udd with actively transcribed rRNA genes. Studies on mammalian cells show that rDNA repeats in the nucleolus form loops connecting promoter and termination regions (91,92) or more complex structures, in which the transcribed rDNA region is wrapped around the core formed by SL1 (93). Hence, it is conceivable that in ChIP experiments some cross-linking can occur between proteins associated with the promoter and pre-rRNA-coding part of the same rDNA unit. Thus, our results fit well with the idea that Udd being a component of the Pol I transcription promoting apparatus is associated mainly with intact rDNA units. Overall, we suggest that SL1-like indirectly controls the repression of inserted rRNA genes possibly through redistribution of Pol I toward uninserted rDNA repeats (see Discussion for details). We also found Udd enrichment in the region of rDNA transcription termination on the border of 28S and IGS sequences, as well as in the IGS (Figure 6A and Supplementary Figure S9). This can be interpreted as the formation of a spatial loop or as a binding of the SL1-like to putative IGS promoters. A similar pattern of Udd distribution along rDNA units was observed in both ovaries and carcasses, whereas enrichment levels were lower in carcasses (Supplementary Figure S9), which may reflect a higher rate of rRNA synthesis in ovarian cells.

### H3K9me3 level depends on transcriptional status of rDNA repeats

Next we tested whether derepression of R2-inserted rDNA units in *udd* mutants was accompanied by changes in repressive chromatin marks. ChIP-qPCR revealed 2- to 3-fold reduction of H3K9me3 occupancy at R2 elements and rDNA promoter regions in both *udd^1^*/*udd^null^* and *udd^0683-G4^*/*udd^null^* ovaries when compared to control heterozygotes (Figure 6C). The H3K9me3 levels at uninserted 28S were also decreased, though to a lesser extent than at the R2 sequence (Figure 6C). This result suggests that some rRNA genes lacking R1/R2 insertions are also normally silenced and heterochromatinized, whereas in *udd* mutants they may be transcriptionally activated and lose heterochromatin marks in a similar way to R2-inserted rDNA units. *udd* mutations had no effect on the H3K9me3 occupancy at telomeric HeT-A elements (Figure 6С) and other heterochromatic genome regions, such as piRNA clusters (data not shown), indicating that Udd affects the H3K9me3 level specifically within rDNA locus. We also observed a reduction of HP1a in the chromatin associated with both R2 insertions and uninserted 28S rDNA sequences in *udd^1^*/*udd^null^* ovaries (Supplementary Figure S10A). However, the levels of H3K27me3 and H4K20me3 marks, which have also been shown to be involved in rDNA silencing in mammalian cells (94,95) did not change significantly for any of the analyzed sequences (Supplementary Figure S10B and C).

Our observations suggest that the reduction of the H3K9me3 level in rRNA genes in the *udd* mutant ovaries can be caused by their transcriptional activation, and vice versa repressive chromatin marks can normally be recruited to rDNA units as a consequence of their transcriptional silencing. To additionally clarify a relationship between the rDNA transcriptional status and heterochromatin marks, we examined the rDNA chromatin after inhibition of transcription by actinomycin D (ActD) during 2 h in OSC cultured cells. ActD nonspecifically blocks all types of RNA polymerase (96). We detected cessation of rRNA synthesis in ActD-treated cells, which was accompanied by a reduction of a nucleolar area occupied by fibrillarin (Supplementary Figure S11A and B) but did not lead to cell death and block of DNA replication (Supplementary Figure S11C and D). Interestingly, Udd was largely released from the nucleolus and found in the cytoplasm of ActD-treated cells (Supplementary Figure S11A). ChIP-qPCR revealed a loss of Udd binding at the rDNA promoter, terminator, and IGS regions where Udd is enriched in untreated cells (Figure 6D) additionally indicating that Udd interacts with actively transcribed rRNA genes. ChIP-qPCR analysis demonstrated a significant increase of H3K9me3 mark at uninserted 28S rDNA sequences and rDNA promoter regions but not at R2 insertions in ActD-treated cells compared to control (Figure 6E). Thus, repressive chromatin can be rapidly established on transcriptionally inactive rDNA units that supports the notion that the difference in H3K9me3 levels between inserted and intact rRNA genes can be a consequence of their distinct transcriptional states.

## DISCUSSION

### Possible mechanisms of discrimination between interrupted and intact ribosomal genes and activation of R2 element

It has been known for several decades that a substantial number of rDNA units in the *D. melanogaster* genome contain insertions of R1 and R2 retrotransposons usually causing these units to be expressed at very low levels (47,48,51). The R2 element integrates exclusively into the rDNA locus and is capable to be transcribed only as part of pre-rRNA (39–42). Here we found that the impairment of the putative Pol I transcription initiation complex SL1-like abolished silencing of interrupted rDNA units and especially those with insertions of R2 (Figure 3). *Drosophila* SL1-like was described as an analog of the mammalian SL1 complex (68), which promotes rDNA transcription by recognizing and binding the rDNA core promoter sequence and recruiting Pol I (87). Three components of SL1-like have been identified: TAF1b and TAF1c-like subunits, which have orthologs in mammals, and a *Drosophila*-specific small subunit Udd (68). Depletion of any of the three components induced upregulation of interrupted rRNA genes (Figure 3A). Apparently, the complete loss of these proteins is lethal, and the analyzed effects are caused by a partial impairment of the SL1-like complex. In contrast to the previous report (68), we did not find a dramatic decrease in the total pre-rRNA derived from both interrupted and intact rDNA units in the ovaries lacking SL1-like components, except for a 2-fold reduction in *udd^1^*/*udd^null^* mutants (Figure 4, Supplementary Figure S7). However, we assume that transcription of intact rRNA genes may decrease to some extent upon all examined mutations and knockdowns of SL1-like subunits.

Activation of interrupted rDNA units upon *udd* mutations occurs predominantly in germ cells (Figure 3B,C). This effect may be attributed to the fact that a small amount of Udd retained in *udd^1^* and *udd^0683-G4^* hypomorphs is sufficient to ensure Udd function in somatic cells. Nurse cells require much more Pol I transcription factors, because provide an especially high level of rRNA synthesis. It has been calculated that a stage 14 oocyte contains approximately 2 × 10^10^ ribosomes (66), the vast majority of which are produced in the nurse cells during stages 7 through 10 (67). The strongest accumulation of R2 transcripts was observed in *udd^0683-G4^*/*udd^null^* ovaries at these stages (Figure 3C).

The mechanism of repression of inserted rDNA units remains generally unknown. In accord with the previous work (50), we revealed a sharp reduction in the amount of transcripts from the 5′ to the 3′ end of the R2 insertions indicating that transcription is partially interrupted within the insertions (Figure 5A). On the other hand, we found that *udd* mutations caused an increase of transcription along the entire length of the R2 element and the upstream 28S rRNA sequence (Figure 5A). This result suggests that transcription of inserted rDNA units normally can be less efficiently initiated than that of intact ones and this repression is released in *udd* mutants. It is tempting to speculate that transcriptional selection of rDNA units can be established by spatial loops formed between the rDNA promoter and terminator regions, which can contribute to efficient switching from termination to re-initiation of transcription as was demonstrated in mammalian cells (91–93,97). Since transcription usually reaches the terminator in intact rRNA genes, it can be effectively re-initiated at their promoters, whereas in inserted rDNA units transcriptional re-initiation may occur much less frequently due to the often termination within or at the ends of TE insertions. The possible role of spatial interactions in transcriptional selection of rDNA repeats is supported by the R2 upregulation upon mutation of the chromatin architectural protein CTCF (53). Other factors such as deposition of active chromatin marks, co-transcriptional rRNA processing, and phase separation could also be involved in the positive feedback loop, promoting the accumulation of components of the Pol I transcription apparatus on intact rDNA units. On the contrary, silencing of inserted rDNA units can be further enhanced by a repressive regulatory loop, which includes heterochromatin formation and other mechanisms (see the second part of the Discussion).

We consider two non mutually exclusive models that could explain the upregulation of inserted rDNA units upon the impairment of SL1-like function. First, we suppose that the SL1-like complex may facilitate effective transcription re-initiation on active rDNA units or participate in the bridging of the rDNA promoter and terminator, which is consistent with Udd enrichment at both regions (Figure 6A and Supplementary Figure S9). Of note, SL1 in mammalian cells is thought to be involved in anchoring the core promoter, upstream enhancer and terminator elements, which provides a spatial arrangement favorable for active rRNA synthesis (93). Therefore, we hypothesize that the partial SL1-like impairment can abrogate the feedback regulatory loop on intact rRNA genes. Without this loop, Pol I complexes may be indiscriminately redistributed over all rDNA units, including those with insertions. The observed elevated transcription of IGS repeats in *udd* mutants (Figure 4B) can also be attributable to this redistribution since these regions contain promoter elements resembling the main rDNA promoter (98,99). Second, it is conceivable that derepression of normally silent rRNA genes with preferential activation of the R2 element may be induced by a transcriptional reprogramming, which is triggered in response to decreased rRNA synthesis upon the impairment of SL1-like. Under natural conditions, a decline of rRNA production can be caused by a reduction of rDNA copy number, which occurs due to spontaneous intra-chromatid recombination within the rDNA locus (100). The R2 protein is suggested to initiate the restoration of rDNA clusters by generating DNA breaks that induce the recombination-mediated repair (45,46). Thus, transcriptional activation of the R2-inserted rDNA units may serve as an adaptive response in order to produce the R2 protein, which then stimulates the increase of rDNA copy number. This model better interprets an extremely high upregulation of the R2 element observed in *udd^0683-G4^*/*udd^null^* ovaries. Our assumptions are consistent with previous finding that loss of a ribosome assembly factor Nopp140 leads to both reduced rRNA synthesis (101) and R2 activation (64). Similarly to the effects of the SL1-like impairment, R2 elements displayed preferential induction upon Nopp140 loss as compared to R1 elements (64). Further experiments are needed to test these hypotheses. It would be interesting to explore whether disruption of other components of the Pol I transcription machinery, in particular Tif-IA, influences on selective regulation of rDNA units. Elucidation of how cells are capable to sense the level of rRNA production as well as the mechanism of subsequent transcriptional reprogramming are also of great interest for further exploration. Besides, the follow-up studies may clarify a possible link between these transcriptional changes and the alterations of nucleolar structure observed in *udd* mutant ovaries (Figure 3C–F).

### Heterochromatin marks in the context of *Drosophila* rRNA genes

Silent rRNA genes in mammalian cells are known to have hypermethylated DNA and regular nucleosomes carrying repressive histone modifications, such as H3K9me3, H4K20me3 and H3K27me3 (94,95,102,103) (see (26–29) for reviews). In *D. melanogaster*, heterochromatin components have been shown to be implicated in maintaining the nucleolar structure and preventing recombination between rDNA repeats (22), as well as in nucleolar dominance, a phenomenon whereby an entire rDNA cluster is silenced (104). However, the association of repressive chromatin marks with silencing of individual rDNA units in *Drosophila* was not evident. Here we found higher levels of H3K9me3 and HP1a in the chromatin of inserted rDNA units compared to uninserted ones (Figures 1C, D, and 6C, E). Furthermore, upregulation of R2-inserted rRNA genes in the ovaries of *udd* mutants was accompanied by a reduction of H3K9me3 and HP1a enrichments (Figure 6C and Supplementary Figure S10), whereas inhibition of transcription by ActD led to the acquisition of H3K9me3 at intact rRNA genes (Figure 6E). These results demonstrate that heterochromatin marks indeed are associated with rDNA unit repression in *Drosophila* and support for the idea of chromatin-based differentiation between individual rDNA repeats within one array. Our findings parallel with the results revealing the coexistence of methylated and unmethylated rDNA units within the same cluster using FISH and immunostaining of single DNA fibers in human cells (20). Moreover, the transcriptionally inert rDNA genes associated with HP1 have been recently demonstrated inside active human NORs by super-resolution microscopy (105).

Earlier, based on the psoralen cross-linking assay it was suggested that a substantial part of *Drosophila* rRNA genes lacking R1 and R2 insertions is also silenced (50). This repression may be caused in particular by other defects of rDNA sequences. Presumably, these rDNA units are also marked by repressive chromatin modifications. Therefore, transcriptionally active rRNA genes may in fact have lower levels of H3K9me3 and HP1a than was shown for R1/R2-uninserted rDNA units detected by our ChIP-qPCR analysis. The observed decrease of H3K9me3 mark at uninserted 28S region in *udd* mutants (Figure 6C) can be owing to the derepression of these units.

We showed that the lack of HP1a protein, H3K9 HMTs, as well as H3K9 methylation itself leads to several fold upregulation of R1 and R2 elements in ovaries (Figure 2). Interestingly, Egg and Su(var)3–9 HMTs did not compensate the loss of each other, which may be due to the fact that Egg is involved in *de novo* H3K9me3 establishment, while Su(var)3–9 is required for H3K9me3 spreading (84), and its binding with the chromatin partially depends on Egg in ovarian germ cells (106). The effects of heterochromatin loss observed in our work are quantitatively similar to R2 upregulation revealed previously upon knockdown of histone H1 (62) and upon lamin depletion, which induces a strong decrease of the H3K9me3 level at the R2 element (63). Altogether, we propose that heterochromatin marks are deposited on less effectively transcribed rDNA units, strengthening their repression, whereas transcriptional activation can remove the repressive chromatin modifications (see model in Supplementary Figure S12). The H3K9me3 recruitment to uninserted rDNA repeats upon inhibition of transcription by 2 h ActD treatment demonstrates a possibility of a remarkably rapid heterochromatin formation on transcriptionally inactive rDNA units (Figure 6E).

Other mechanisms of interrupted rRNA gene silencing may include small RNA pathways (siRNA and piRNA), the disruption of which lead to some modest upregulation of R1 and R2 elements (58–61) possibly on the post-transcriptional level. At the same time, the loss of piRNA-binding protein Piwi on a specific genetic background may cause general transcriptional repression of the rDNA cluster, which leads to partial removal of Udd from the nucleoli of nurse cells (107). In parallel with our investigations, it was shown that knockdowns of SUMO (Small Ubiquitin-like Modifier) and SUMO ligase Ubc9 induce dramatic derepression of inserted rDNA units and, probably, activation of silent rDNA repeats lacking R1/R2 insertions (108). The authors suggest that multiple chromatin proteins associated with silenced rDNA units undergo SUMOylation. Although Udd was found among nucleolar targets of SUMOylation (108), the effects upon SUMO knockdown cannot be explained solely through Udd or SL1-like functions, but apparently represent a more extensive release of rDNA transcription. In particular, the loss of SUMO leads to a stronger derepression of R1 compared to R2 elements and increases the total pre-rRNA production (108). It is likely that the SUMO pathway, independently of heterochromatin, may play a key role in the repressive feedback loop, which maintains the silencing of rDNA units.

As a whole, our study, along with other recent publications, uncovers that transcriptional selection of individual rRNA genes can be regulated by a complex of various molecular mechanisms that are only beginning to be elucidated.

## DATA AVAILABILITY

NGS sequence library datasets have been submitted to the NCBI Gene Expression Omnibus (GEO) (http://www.ncbi.nlm.nih.gov/geo/) under accession number GSE183035.

## Supplementary Material

gkab1276_Supplemental_Files

## References

[1] Russell J. , Zomerdijk J.C. RNA-polymerase-I-directed rDNA transcription, life and works. Trends Biochem. Sci. 2005; 30:87–96. 15691654 10.1016/j.tibs.2004.12.008 PMC3858833

[2] Grummt I. Life on a planet of its own: regulation of RNA polymerase i transcription in the nucleolus. Genes Dev. 2003; 17:1691–1702. 12865296 10.1101/gad.1098503R

[3] Moss T. , Stefanovsky V.Y. At the center of eukaryotic life. Cell. 2002; 109:545–548. 12062097 10.1016/s0092-8674(02)00761-4

[4] Warner J.R. The economics of ribosome biosynthesis in yeast. Trends Biochem. Sci. 1999; 24:437–440. 10542411 10.1016/s0968-0004(99)01460-7

[5] McStay B. Nucleolar organizer regions: genomic ‘dark matter’ requiring illumination. Genes Dev. 2016; 30:1598–1610. 27474438 10.1101/gad.283838.116 PMC4973289

[6] Miller O.L. , Beatty B.R Visualization of nucleolar genes. Science. 1969; 164:955–957. 5813982 10.1126/science.164.3882.955

[7] McKnight S.L. , Miller O.L. Jr Ultrastructural patterns of RNA synthesis during early embryogenesis of drosophila melanogaster. Cell. 1976; 8:305–319. 822943 10.1016/0092-8674(76)90014-3

[8] Conconi A. , Widmer R.M., Koller T., Sogo J.M. Two different chromatin structures coexist in ribosomal RNA genes throughout the cell cycle. Cell. 1989; 57:753–761. 2720786 10.1016/0092-8674(89)90790-3

[9] Conconi A. , Sogo J.M., Ryan C.A. Ribosomal gene clusters are uniquely proportioned between open and closed chromatin structures in both tomato leaf cells and exponentially growing suspension cultures. Proc. Natl. Acad. Sci. USA. 1992; 89:5256–5260. 11607297 10.1073/pnas.89.12.5256 PMC49270

[10] Stancheva I. , Lucchini R., Koller T., Sogo J.M. Chromatin structure and methylation of rat rRNA genes studied by formaldehyde fixation and psoralen cross-linking. Nucleic Acids Res. 1997; 25:1727–1735. 9108154 10.1093/nar/25.9.1727 PMC146648

[11] Dammann R. , Lucchini R., Koller T., Sogo J.M. Chromatin structures and transcription of rDNA in yeast saccharomyces cerevisiae. Nucleic Acids Res. 1993; 21:2331–2338. 8506130 10.1093/nar/21.10.2331 PMC309528

[12] Parks M.M. , Kurylo C.M., Dass R.A., Bojmar L., Lyden D., Vincent C.T., Blanchard S.C. Variant ribosomal RNA alleles are conserved and exhibit tissue-specific expression. Sci. Adv. 2018; 4:eaao0665. 29503865 10.1126/sciadv.aao0665 PMC5829973

[13] Pontvianne F. , Blevins T., Chandrasekhara C., Mozgova I., Hassel C., Pontes O.M., Tucker S., Mokros P., Muchova V., Fajkus J. et al. Subnuclear partitioning of rRNA genes between the nucleolus and nucleoplasm reflects alternative epiallelic states. Genes Dev. 2013; 27:1545–1550. 23873938 10.1101/gad.221648.113 PMC3731543

[14] Rabanal F.A. , Mandakova T., Soto-Jimenez L.M., Greenhalgh R., Parrott D.L., Lutzmayer S., Steffen J.G., Nizhynska V., Mott R., Lysak M.A. et al. Epistatic and allelic interactions control expression of ribosomal RNA gene clusters in arabidopsis thaliana. Genome Biol. 2017; 18:75. 28464948 10.1186/s13059-017-1209-z PMC5414317

[15] Tucker S. , Vitins A., Pikaard C.S. Nucleolar dominance and ribosomal RNA gene silencing. Curr. Opin. Cell Biol. 2010; 22:351–356. 20392622 10.1016/j.ceb.2010.03.009 PMC2912983

[16] Schlesinger S. , Selig S., Bergman Y., Cedar H. Allelic inactivation of rDNA loci. Genes Dev. 2009; 23:2437–2447. 19833769 10.1101/gad.544509 PMC2764490

[17] Roussel P. , Andre C., Comai L., Hernandez-Verdun D The rDNA transcription machinery is assembled during mitosis in active NORs and absent in inactive NORs. J. Cell Biol. 1996; 133:235–246. 8609158 10.1083/jcb.133.2.235 PMC2120807

[18] Greil F. , Ahmad K. Nucleolar dominance of the y chromosome in drosophila melanogaster. Genetics. 2012; 191:1119–1128. 22649076 10.1534/genetics.112.141242 PMC3415996

[19] Kim J.H. , Dilthey A.T., Nagaraja R., Lee H.S., Koren S., Dudekula D., Wood Iii W.H., Piao Y., Ogurtsov A.Y., Utani K. et al. Variation in human chromosome 21 ribosomal RNA genes characterized by TAR cloning and long-read sequencing. Nucleic Acids Res. 2018; 46:6712–6725. 29788454 10.1093/nar/gky442 PMC6061828

[20] Zillner K. , Komatsu J., Filarsky K., Kalepu R., Bensimon A., Nemeth A. Active human nucleolar organizer regions are interspersed with inactive rDNA repeats in normal and tumor cells. Epigenomics. 2015; 7:363–378. 26077426 10.2217/epi.14.93

[21] McStay B. Nucleolar dominance: a model for rRNA gene silencing. Genes Dev. 2006; 20:1207–1214. 16702398 10.1101/gad.1436906

[22] Peng J.C. , Karpen G.H. H3K9 methylation and RNA interference regulate nucleolar organization and repeated DNA stability. Nat. Cell Biol. 2007; 9:25–35. 17159999 10.1038/ncb1514 PMC2819265

[23] Guetg C. , Lienemann P., Sirri V., Grummt I., Hernandez-Verdun D., Hottiger M.O., Fussenegger M., Santoro R. The NoRC complex mediates the heterochromatin formation and stability of silent rRNA genes and centromeric repeats. EMBO J. 2010; 29:2135–2146. 20168299 10.1038/emboj.2010.17 PMC2905252

[24] Kobayashi T. Ribosomal RNA gene repeats, their stability and cellular senescence. Proc. Jpn. Acad. Ser. B Phys. Biol. Sci. 2014; 90:119–129. 10.2183/pjab.90.119 PMC4055705 24727936

[25] Caburet S. , Conti C., Schurra C., Lebofsky R., Edelstein S.J., Bensimon A. Human ribosomal RNA gene arrays display a broad range of palindromic structures. Genome Res. 2005; 15:1079–1085. 16024823 10.1101/gr.3970105 PMC1182220

[26] Schofer C. , Weipoltshammer K. Nucleolus and chromatin. Histochem. Cell Biol. 2018; 150:209–225. 30046888 10.1007/s00418-018-1696-3 PMC6096769

[27] Hamperl S. , Wittner M., Babl V., Perez-Fernandez J., Tschochner H., Griesenbeck J. Chromatin states at ribosomal DNA loci. Biochim. Biophys. Acta. 2013; 1829:405–417. 23291532 10.1016/j.bbagrm.2012.12.007

[28] Srivastava R. , Srivastava R., Ahn S.H. The epigenetic pathways to ribosomal DNA silencing. Microbiol. Mol. Biol. Rev. 2016; 80:545–563. 27250769 10.1128/MMBR.00005-16 PMC4981667

[29] Grummt I. , Langst G. Epigenetic control of RNA polymerase i transcription in mammalian cells. Biochim. Biophys. Acta. 2013; 1829:393–404. 23063748 10.1016/j.bbagrm.2012.10.004

[30] Eickbush T.H. , Eickbush D.G. Integration, regulation, and long-term stability of R2 retrotransposons. Microbiol. Spectr. 2015; 3:MDNA3-0011-2014. 10.1128/microbiolspec.MDNA3-0011-2014 PMC4498411 26104703

[31] DiMario P. , James A., Raje H. Proteins of the Nucleolus. 2013; Dordrecht Springer 39–78.

[32] Ritossa F.M. , Atwood K.C., Spiegelman S. A molecular explanation of the bobbed mutants of drosophila as partial deficiencies of “ribosomal” DNA. Genetics. 1966; 54:819–834. 5970623 10.1093/genetics/54.3.819 PMC1211204

[33] Jakubczak J.L. , Zenni M.K., Woodruff R.C., Eickbush T.H. Turnover of R1 (type I) and R2 (type II) retrotransposable elements in the ribosomal DNA of drosophila melanogaster. Genetics. 1992; 131:129–142. 1317313 10.1093/genetics/131.1.129 PMC1204947

[34] Jakubczak J.L. , Burke W.D., Eickbush T.H. Retrotransposable elements R1 and R2 interrupt the rRNA genes of most insects. Proc. Natl. Acad. Sci. USA. 1991; 88:3295–3299. 1849649 10.1073/pnas.88.8.3295 PMC51433

[35] Kidd S.J. , Glover D.M. A DNA segment from d. melanogaster which contains five tandemly repeating units homologous to the major rDNA insertion. Cell. 1980; 19:103–119. 6244098 10.1016/0092-8674(80)90392-x

[36] Peacock W.J. , Appels R., Endow S., Glover D Chromosomal distribution of the major insert in drosophila melanogaster 28S rRNA genes. Genet. Res. 1981; 37:209–214. 6266913 10.1017/s0016672300020176

[37] Browne M.J. , Read C.A., Roiha H., Glover D.M. Site specific insertion of a type i rDNA element into a unique sequence in the drosophila melanogaster genome. Nucleic Acids Res. 1984; 12:9111–9122. 6096818 10.1093/nar/12.23.9111 PMC320441

[38] Plata M.P. , Kang H.J., Zhang S., Kuruganti S., Hsu S.J., Labrador M. Changes in chromatin structure correlate with transcriptional activity of nucleolar rDNA in polytene chromosomes. Chromosoma. 2009; 118:303–322. 19066928 10.1007/s00412-008-0198-9

[39] George J.A. , Eickbush T.H. Conserved features at the 5 end of drosophila R2 retrotransposable elements: implications for transcription and translation. Insect Mol. Biol. 1999; 8:3–10. 9927169 10.1046/j.1365-2583.1999.810003.x

[40] Eickbush D.G. , Ye J., Zhang X., Burke W.D., Eickbush T.H. Epigenetic regulation of retrotransposons within the nucleolus of drosophila. Mol. Cell. Biol. 2008; 28:6452–6461. 18678644 10.1128/MCB.01015-08 PMC2577413

[41] Eickbush D.G. , Eickbush T.H. Transcription of endogenous and exogenous R2 elements in the rRNA gene locus of drosophila melanogaster. Mol. Cell. Biol. 2003; 23:3825–3836. 12748285 10.1128/MCB.23.11.3825-3836.2003 PMC155226

[42] Eickbush D.G. , Eickbush T.H. R2 retrotransposons encode a self-cleaving ribozyme for processing from an rRNA cotranscript. Mol. Cell. Biol. 2010; 30:3142–3150. 20421411 10.1128/MCB.00300-10 PMC2897577

[43] Jamburuthugoda V.K. , Eickbush T.H. Identification of RNA binding motifs in the R2 retrotransposon-encoded reverse transcriptase. Nucleic Acids Res. 2014; 42:8405–8415. 24957604 10.1093/nar/gku514 PMC4117753

[44] Yang J. , Malik H.S., Eickbush T.H. Identification of the endonuclease domain encoded by R2 and other site-specific, non-long terminal repeat retrotransposable elements. Proc. Natl. Acad. Sci. USA. 1999; 96:7847–7852. 10393910 10.1073/pnas.96.14.7847 PMC22150

[45] Hawley R.S. , Marcus C.H. Recombinational controls of rDNA redundancy in drosophila. Annu. Rev. Genet. 1989; 23:87–120. 2694947 10.1146/annurev.ge.23.120189.000511

[46] Nelson J.O. , Slicko A., Yamashita Y.M. The retrotransposon R2 maintains drosophila ribosomal DNA repeats. 2021; biorXiv doi:12 July 2021, preprint: not peer reviewed 10.1101/2021.07.12.451825. PMC10266012 37252996

[47] Long E.O. , Dawid I.B. Expression of ribosomal DNA insertions in drosophila melanogaster. Cell. 1979; 18:1185–1196. 117903 10.1016/0092-8674(79)90231-9

[48] Jolly D.J. , Thomas C.A. Jr Nuclear RNA transcripts from drosophila melanogaster ribosomal RNA genes containing introns. Nucleic Acids Res. 1980; 8:67–84. 6243780 10.1093/nar/8.1.67 PMC327243

[49] Kidd S.J. , Glover D.M. Drosophila melanogaster ribosomal DNA containing type II insertions is variably transcribed in different strains and tissues. J. Mol. Biol. 1981; 151:645–662. 6276565 10.1016/0022-2836(81)90428-9

[50] Ye J. , Eickbush T.H. Chromatin structure and transcription of the R1- and R2-inserted rRNA genes of drosophila melanogaster. Mol. Cell. Biol. 2006; 26:8781–8790. 17000772 10.1128/MCB.01409-06 PMC1636831

[51] Jamrich M. , Miller O.L. Jr The rare transcripts of interrupted rRNA genes in drosophila melanogaster are processed or degraded during synthesis. EMBO J. 1984; 3:1541–1545. 6430695 10.1002/j.1460-2075.1984.tb02008.x PMC557556

[52] Glatzer K.H. Lengths of transcribed rDNA repeating units in spermatocytes of drosophila hydei: only genes without an intervening sequence are expressed. Chromosoma. 1979; 75:161–175. 533667 10.1007/BF00292205

[53] Guerrero P.A. , Maggert K.A. The CCCTC-binding factor (CTCF) of drosophila contributes to the regulation of the ribosomal DNA and nucleolar stability. PLoS One. 2011; 6:e16401. 21283722 10.1371/journal.pone.0016401 PMC3024428

[54] Raje H.S. , Lieux M.E., DiMario P.J. R1 retrotransposons in the nucleolar organizers of drosophila melanogaster are transcribed by RNA polymerase i upon heat shock. Transcription. 2018; 9:273–285. 30063880 10.1080/21541264.2018.1506682 PMC6150621

[55] Merel V. , Boulesteix M., Fablet M., Vieira C. Transposable elements in drosophila. Mob DNA. 2020; 11:23. 32636946 10.1186/s13100-020-00213-z PMC7334843

[56] Czech B. , Hannon G.J. One loop to rule them all: the ping-pong cycle and piRNA-Guided silencing. Trends Biochem. Sci. 2016; 41:324–337. 26810602 10.1016/j.tibs.2015.12.008 PMC4819955

[57] Ozata D.M. , Gainetdinov I., Zoch A., O’Carroll D., Zamore P.D. PIWI-interacting RNAs: small RNAs with big functions. Nat. Rev. Genet. 2019; 20:89–108. 30446728 10.1038/s41576-018-0073-3

[58] Li W. , Prazak L., Chatterjee N., Gruninger S., Krug L., Theodorou D., Dubnau J. Activation of transposable elements during aging and neuronal decline in drosophila. Nat. Neurosci. 2013; 16:529–531. 23563579 10.1038/nn.3368 PMC3821974

[59] Jones B.C. , Wood J.G., Chang C., Tam A.D., Franklin M.J., Siegel E.R., Helfand S.L. A somatic piRNA pathway in the drosophila fat body ensures metabolic homeostasis and normal lifespan. Nat. Commun. 2016; 7:13856. 28000665 10.1038/ncomms13856 PMC5187580

[60] Perrat P.N. , DasGupta S., Wang J., Theurkauf W., Weng Z., Rosbash M., Waddell S. Transposition-driven genomic heterogeneity in the drosophila brain. Science. 2013; 340:91–95. 23559253 10.1126/science.1231965 PMC3887341

[61] Mikhaleva E.A. , Leinsoo T.A., Ishizu H., Gvozdev V.A., Klenov M.S. The nucleolar transcriptome regulates piwi shuttling between the nucleolus and the nucleoplasm. Chromosome Res. 2019; 27:141–152. 30539407 10.1007/s10577-018-9595-y

[62] Vujatovic O. , Zaragoza K., Vaquero A., Reina O., Bernues J., Azorin F. Drosophila melanogaster linker histone dH1 is required for transposon silencing and to preserve genome integrity. Nucleic Acids Res. 2012; 40:5402–5414. 22406835 10.1093/nar/gks224 PMC3384340

[63] Chen H. , Zheng X., Xiao D., Zheng Y. Age-associated de-repression of retrotransposons in the drosophila fat body, its potential cause and consequence. Aging Cell. 2016; 15:542–552. 27072046 10.1111/acel.12465 PMC4854910

[64] He F. , James A., Raje H., Ghaffari H., DiMario P. Deletion of drosophila nopp140 induces subcellular ribosomopathies. Chromosoma. 2015; 124:191–208. 25384888 10.1007/s00412-014-0490-9

[65] Zhou J. , Eickbush T.H. The pattern of R2 retrotransposon activity in natural populations of drosophila simulans reflects the dynamic nature of the rDNA locus. PLos Genet. 2009; 5:e1000386. 19229317 10.1371/journal.pgen.1000386 PMC2637433

[66] Klug W.S. , King R.C., Wattiaux J.M. Oogenesis in the suppressor of hairy-wing mutant of drosophila melanogaster. II. Nucleolar morphology and in vitro studies of RNA protein synthesis. J. Exp. Zool. 1970; 174:125–140. 5463647 10.1002/jez.1401740203

[67] Dapples C.C. , King R.C. The development of the nucleolus of the ovarian nurse cell of drosophila melanogaster. Z. Zellforsch. Mikrosk. Anat. 1970; 103:34–47. 5460854 10.1007/BF00335399

[68] Zhang Q. , Shalaby N.A., Buszczak M. Changes in rRNA transcription influence proliferation and cell fate within a stem cell lineage. Science. 2014; 343:298–301. 24436420 10.1126/science.1246384 PMC4084784

[69] Klenov M.S. , Sokolova O.A., Yakushev E.Y., Stolyarenko A.D., Mikhaleva E.A., Lavrov S.A., Gvozdev V.A. Separation of stem cell maintenance and transposon silencing functions of piwi protein. Proc. Natl. Acad. Sci. USA. 2011; 108:18760–18765. 22065765 10.1073/pnas.1106676108 PMC3219103

[70] Klenov M.S. , Lavrov S.A., Korbut A.P., Stolyarenko A.D., Yakushev E.Y., Reuter M., Pillai R.S., Gvozdev V.A. Impact of nuclear piwi elimination on chromatin state in drosophila melanogaster ovaries. Nucleic Acids Res. 2014; 42:6208–6218. 24782529 10.1093/nar/gku268 PMC4041442

[71] Penke T.J. , McKay D.J., Strahl B.D., Matera A.G., Duronio R.J. Direct interrogation of the role of H3K9 in metazoan heterochromatin function. Genes Dev. 2016; 30:1866–1880. 27566777 10.1101/gad.286278.116 PMC5024684

[72] Chanas G. , Lavrov S., Iral F., Cavalli G., Maschat F. Engrailed and polyhomeotic maintain posterior cell identity through cubitus-interruptus regulation. Dev. Biol. 2004; 272:522–535. 15282166 10.1016/j.ydbio.2004.05.020

[73] Maniatis T. , Fritsch E.F., Sambrook J. Molecular cloning : a laboratory manual. 1982; NY Cold Spring Harbor Laboratory.

[74] Le Thomas A. , Rogers A.K., Webster A., Marinov G.K., Liao S.E., Perkins E.M., Hur J.K., Aravin A.A., Toth K.F Piwi induces piRNA-guided transcriptional silencing and establishment of a repressive chromatin state. Genes Dev. 2013; 27:390–399. 23392610 10.1101/gad.209841.112 PMC3589556

[75] Iwasaki Y.W. , Murano K., Ishizu H., Shibuya A., Iyoda Y., Siomi M.C., Siomi H., Saito K. Piwi modulates chromatin accessibility by regulating multiple factors including histone H1 to repress transposons. Mol. Cell. 2016; 63:408–419. 27425411 10.1016/j.molcel.2016.06.008

[76] ElMaghraby M.F. , Andersen P.R., Puhringer F., Hohmann U., Meixner K., Lendl T., Tirian L., Brennecke J. A heterochromatin-specific RNA export pathway facilitates piRNA production. Cell. 2019; 178:964–979. 31398345 10.1016/j.cell.2019.07.007

[77] Patro R. , Duggal G., Love M.I., Irizarry R.A., Kingsford C. Salmon provides fast and bias-aware quantification of transcript expression. Nat. Methods. 2017; 14:417–419. 28263959 10.1038/nmeth.4197 PMC5600148

[78] Sokolova O.A. , Ilyin A.A., Poltavets A.S., Nenasheva V.V., Mikhaleva E.A., Shevelyov Y.Y., Klenov M.S. Yb body assembly on the flamenco piRNA precursor transcripts reduces genic piRNA production. Mol. Biol. Cell. 2019; 30:1544–1554. 30943101 10.1091/mbc.E17-10-0591 PMC6724695

[79] Ilyin A.A. , Ryazansky S.S., Doronin S.A., Olenkina O.M., Mikhaleva E.A., Yakushev E.Y., Abramov Y.A., Belyakin S.N., Ivankin A.V., Pindyurin A.V. et al. Piwi interacts with chromatin at nuclear pores and promiscuously binds nuclear transcripts in drosophila ovarian somatic cells. Nucleic Acids Res. 2017; 45:7666–7680. 28472469 10.1093/nar/gkx355 PMC5570135

[80] Sokolova O.A. , Mikhaleva E.A., Kharitonov S.L., Abramov Y.A., Gvozdev V.A., Klenov M.S. Special vulnerability of somatic niche cells to transposable element activation in drosophila larval ovaries. Sci. Rep. 2020; 10:1076. 31974416 10.1038/s41598-020-57901-2 PMC6978372

[81] Osouda S. , Nakamura Y., de Saint Phalle B., McConnell M., Horigome T., Sugiyama S., Fisher P.A., Furukawa K. Null mutants of drosophila B-type lamin dm(0) show aberrant tissue differentiation rather than obvious nuclear shape distortion or specific defects during cell proliferation. Dev. Biol. 2005; 284:219–232. 15996653 10.1016/j.ydbio.2005.05.022

[82] Eissenberg J.C. , Elgin S.C.R. HP1a: a structural chromosomal protein regulating transcription. Trends Genet. 2014; 30:103–110. 24555990 10.1016/j.tig.2014.01.002 PMC3991861

[83] Lu X. , Wontakal S.N., Kavi H., Kim B.J., Guzzardo P.M., Emelyanov A.V., Xu N., Hannon G.J., Zavadil J., Fyodorov D.V. et al. Drosophila H1 regulates the genetic activity of heterochromatin by recruitment of Su(var)3-9. Science. 2013; 340:78–81. 23559249 10.1126/science.1234654 PMC3756538

[84] Sienski G. , Batki J., Senti K.A., Donertas D., Tirian L., Meixner K., Brennecke J. Silencio/CG9754 connects the Piwi-piRNA complex to the cellular heterochromatin machinery. Genes Dev. 2015; 29:2258–2271. 26494711 10.1101/gad.271908.115 PMC4647559

[85] Brower-Toland B. , Riddle N.C., Jiang H., Huisinga K.L., Elgin S.C. Multiple SET methyltransferases are required to maintain normal heterochromatin domains in the genome of drosophila melanogaster. Genetics. 2009; 181:1303–1319. 19189944 10.1534/genetics.108.100271 PMC2666501

[86] Lundberg L.E. , Stenberg P., Larsson J. HP1a, Su(var)3-9, SETDB1 and POF stimulate or repress gene expression depending on genomic position, gene length and expression pattern in drosophila melanogaster. Nucleic Acids Res. 2013; 41:4481–4494. 23476027 10.1093/nar/gkt158 PMC3632140

[87] Goodfellow S.J. , Zomerdijk J.C. Basic mechanisms in RNA polymerase i transcription of the ribosomal RNA genes. Subcell. Biochem. 2013; 61:211–236. 23150253 10.1007/978-94-007-4525-4_10 PMC3855190

[88] Grewal S.S. , Evans J.R., Edgar B.A. Drosophila TIF-IA is required for ribosome synthesis and cell growth and is regulated by the TOR pathway. J. Cell Biol. 2007; 179:1105–1113. 18086911 10.1083/jcb.200709044 PMC2140016

[89] Marygold S.J. , Roote J., Reuter G., Lambertsson A., Ashburner M., Millburn G.H., Harrison P.M., Yu Z., Kenmochi N., Kaufman T.C. et al. The ribosomal protein genes and minute loci of drosophila melanogaster. Genome Biol. 2007; 8:R216. 17927810 10.1186/gb-2007-8-10-r216 PMC2246290

[90] Spradling A. Bate M. , Arias A.M. Developmental genetics of oogenesis. The Development of Drosophila melanogaster. 1993; NY Cold Spring Harbor Laboratory Press 1–70.

[91] Nemeth A. , Guibert S., Tiwari V.K., Ohlsson R., Langst G. Epigenetic regulation of TTF-I-mediated promoter-terminator interactions of rRNA genes. EMBO J. 2008; 27:1255–1265. 18354495 10.1038/emboj.2008.57 PMC2367401

[92] Shiue C.N. , Berkson R.G., Wright A.P. c-Myc induces changes in higher order rDNA structure on stimulation of quiescent cells. Oncogene. 2009; 28:1833–1842. 19270725 10.1038/onc.2009.21

[93] Denissov S. , Lessard F., Mayer C., Stefanovsky V., van Driel M., Grummt I., Moss T., Stunnenberg H.G. A model for the topology of active ribosomal RNA genes. EMBO Rep. 2011; 12:231–237. 21331097 10.1038/embor.2011.8 PMC3059908

[94] Bierhoff H. , Dammert M.A., Brocks D., Dambacher S., Schotta G., Grummt I. Quiescence-induced lncrnas trigger H4K20 trimethylation and transcriptional silencing. Mol. Cell. 2014; 54:675–682. 24768537 10.1016/j.molcel.2014.03.032

[95] Xie W. , Ling T., Zhou Y., Feng W., Zhu Q., Stunnenberg H.G., Grummt I., Tao W. The chromatin remodeling complex NuRD establishes the poised state of rRNA genes characterized by bivalent histone modifications and altered nucleosome positions. Proc. Natl. Acad. Sci. USA. 2012; 109:8161–8166. 22570494 10.1073/pnas.1201262109 PMC3361413

[96] Bensaude O. Inhibiting eukaryotic transcription: which compound to choose? How to evaluate its activity. Transcription. 2011; 2:103–108. 21922053 10.4161/trns.2.3.16172 PMC3173647

[97] Maiser A. , Dillinger S., Längst G., Schermelleh L., Leonhardt H., Nemeth A. Super-resolution in situ analysis of active ribosomal DNA chromatin organization in the nucleolus. Sci. Rep. 2020; 10:7462. 32366902 10.1038/s41598-020-64589-x PMC7198602

[98] Murtif V.L. , Rae P.M. In vivo transcription of rDNA spacers in drosophila. Nucleic Acids Res. 1985; 13:3221–3239. 2987877 10.1093/nar/13.9.3221 PMC341231

[99] Grimaldi G. , Fiorentini P., Di Nocera P.P. Spacer promoters are orientation-dependent activators of pre-rRNA transcription in drosophila melanogaster. Mol. Cell. Biol. 1990; 10:4667–4677. 2117701 10.1128/mcb.10.9.4667-4677.1990 PMC361056

[100] Lu K.L. , Nelson J.O., Watase G.J., Warsinger-Pepe N., Yamashita Y.M. Transgenerational dynamics of rDNA copy number in drosophila male germline stem cells. Elife. 2018; 7:e32421. 29436367 10.7554/eLife.32421 PMC5811208

[101] Neumüller R.A. , Gross T., Samsonova A.A., Vinayagam A., Buckner M., Founk K., Hu Y., Sharifpoor S., Rosebrock A.P., Andrews B. et al. Conserved regulators of nucleolar size revealed by global phenotypic analyses. Sci. Signal. 2013; 6:ra70. 23962978 10.1126/scisignal.2004145 PMC3964804

[102] Zhou Y. , Santoro R., Grummt I. The chromatin remodeling complex NoRC targets HDAC1 to the ribosomal gene promoter and represses RNA polymerase i transcription. EMBO J. 2002; 21:4632–4640. 12198165 10.1093/emboj/cdf460 PMC126197

[103] Santoro R. , Li J., Grummt I. The nucleolar remodeling complex NoRC mediates heterochromatin formation and silencing of ribosomal gene transcription. Nat. Genet. 2002; 32:393–396. 12368916 10.1038/ng1010

[104] Warsinger-Pepe N. , Li D., Yamashita Y.M. Regulation of nucleolar dominance in drosophila melanogaster. Genetics. 2020; 214:991–1004. 32122935 10.1534/genetics.119.302471 PMC7153946

[105] Yao R.W. , Xu G., Wang Y., Shan L., Luan P.F., Wang Y., Wu M., Yang L.Z., Xing Y.H., Yang L. et al. Nascent Pre-rRNA sorting via phase separation drives the assembly of dense fibrillar components in the human nucleolus. Mol. Cell. 2019; 76:767–783. 31540874 10.1016/j.molcel.2019.08.014

[106] Maksimov D.A. , Koryakov D.E. Binding of SU(VAR)3-9 partially depends on SETDB1 in the chromosomes of drosophila melanogaster. Cells. 2019; 8:1030. 10.3390/cells8091030 PMC6769583 31491894

[107] Fefelova E.A. , Stolyarenko A.D., Yakushev E.Y., Gvozdev V.A., Klenov M.S. Participation of the piRNA pathway in recruiting a component of RNA polymerase i transcription initiation complex to germline cell nucleoli. Mol. Biol. (Mosk). 2017; 51:824–830. 29116069 10.7868/S0026898417050093

[108] Luo Y. , Fefelova E., Ninova M., Chen Y.A., Aravin A.A. Repression of interrupted and intact rDNA by the SUMO pathway in drosophila melanogaster. Elife. 2020; 9:e52416. 33164748 10.7554/eLife.52416 PMC7676866

