# Supplementary material for: Impaired function of rDNA transcription initiation machinery leads to derepression of ribosomal genes with insertions of R2 retrotransposon
Source: Nucleic Acids Res. 2022 Jan 17;50(2):867–84. doi: 10.1093/nar/gkab1276 (PMC8789037; doi:10.1093/nar/gkab1276)
Supplement: gkab1276_Supplemental_Files [file gkab1276_supplemental_files.zip › Supplementary figures.pdf]

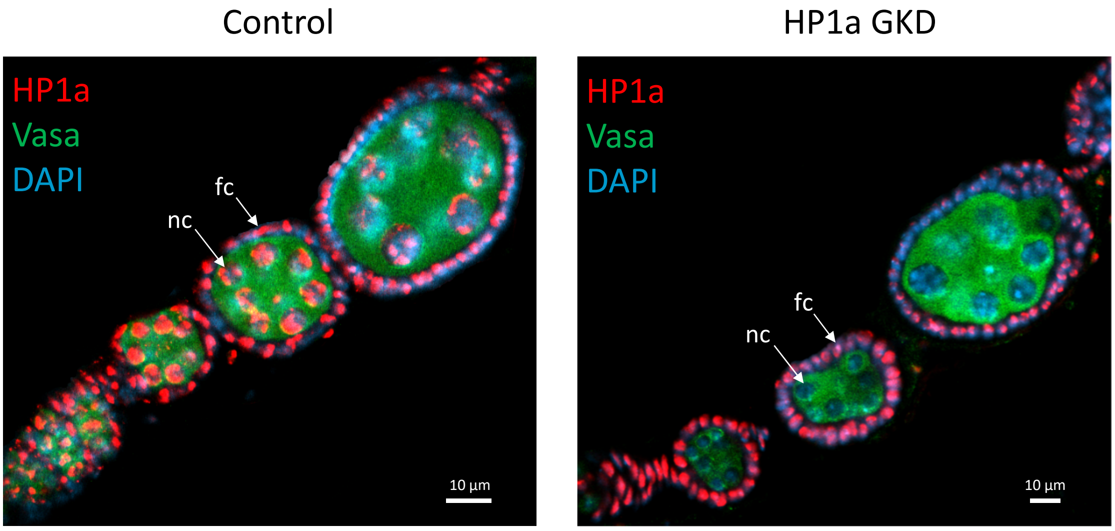

Supplementary Figure S1. Ovarioles of control and nos-GAL4 driven germline knockdown (GKD) of HP1a immunostained for HP1a (red), germ cell marker Vasa (green) and DAPI. Examples of germline nurse cells and somatic follicle cells are indicated as “nc” and “fc”, respectively.

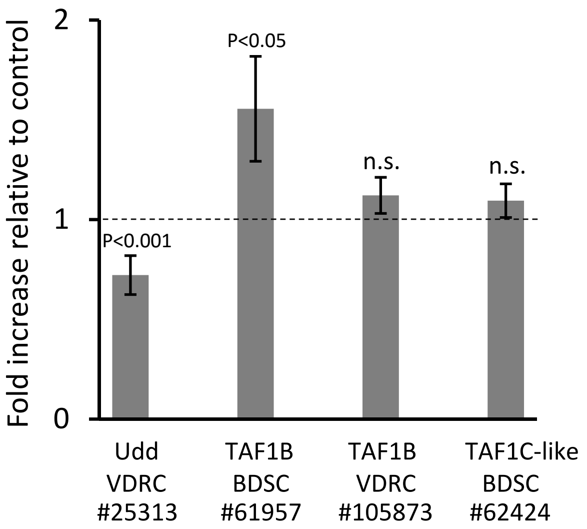

Supplementary Figure S2. Quantification of the pre-rRNA level (18S-ITS1 cotranscripts) by RT-qPCR in ovaries upon nos-GAL4 driven GKD of Udd, TAF1B and TAF1C-like SL1-like subunits. No. of corresponding UAS-RNAi stocks are indicated. Mean fold increase +/− s.d. relative to the control normalized on the rp49 transcript levels are indicated. p-values are based on Student's t-test, n.s. = not significant.

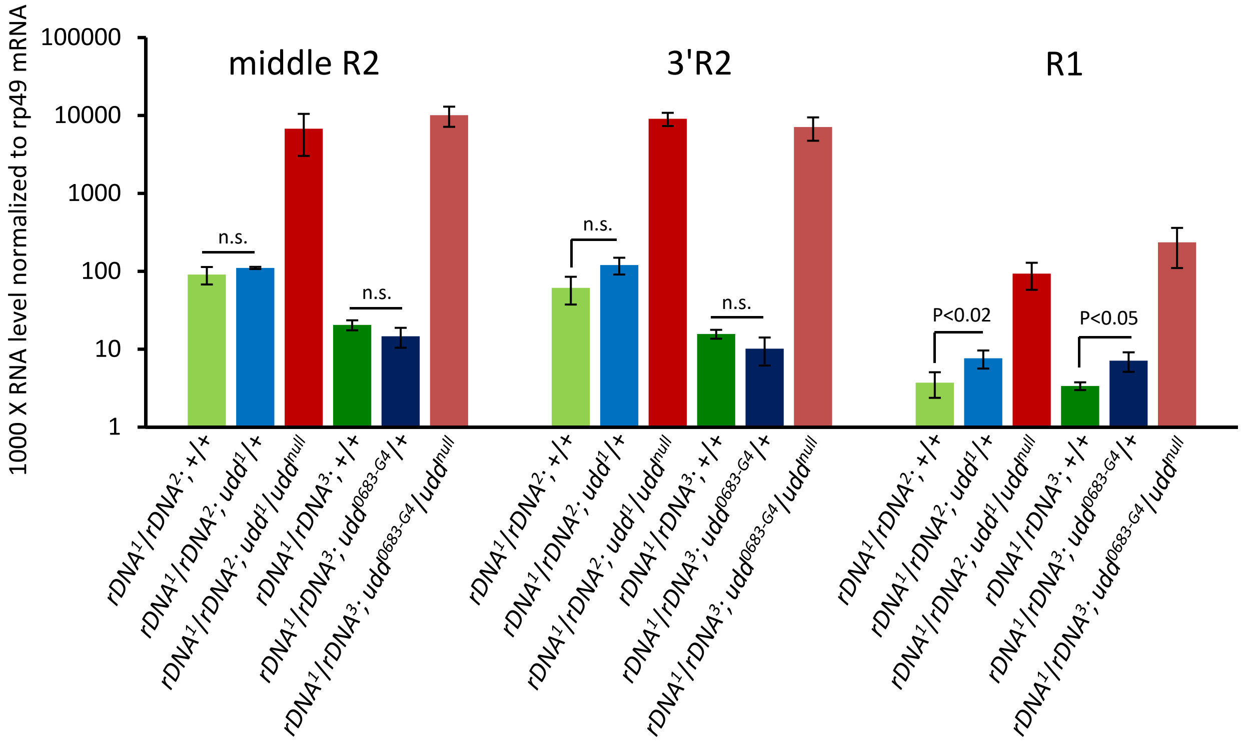

Supplementary Figure S3. *udd* mutants exhibit no pronounced haploinsufficiency. RT-qPCR analysis of R1 and R2 elements in ovaries of *udd* trans-heterozygotes, heterozygotes and flies lacking *udd* mutations (+/+) carrying the same rDNA clusters on X-chromosomes (designated as $rDNA^1/rDNA^2$ and $rDNA^1/rDNA^3$). 1000 X RNA level normalized to rp49 mRNA is shown. p-values are based on Student's t-test, n.s. = not significant.

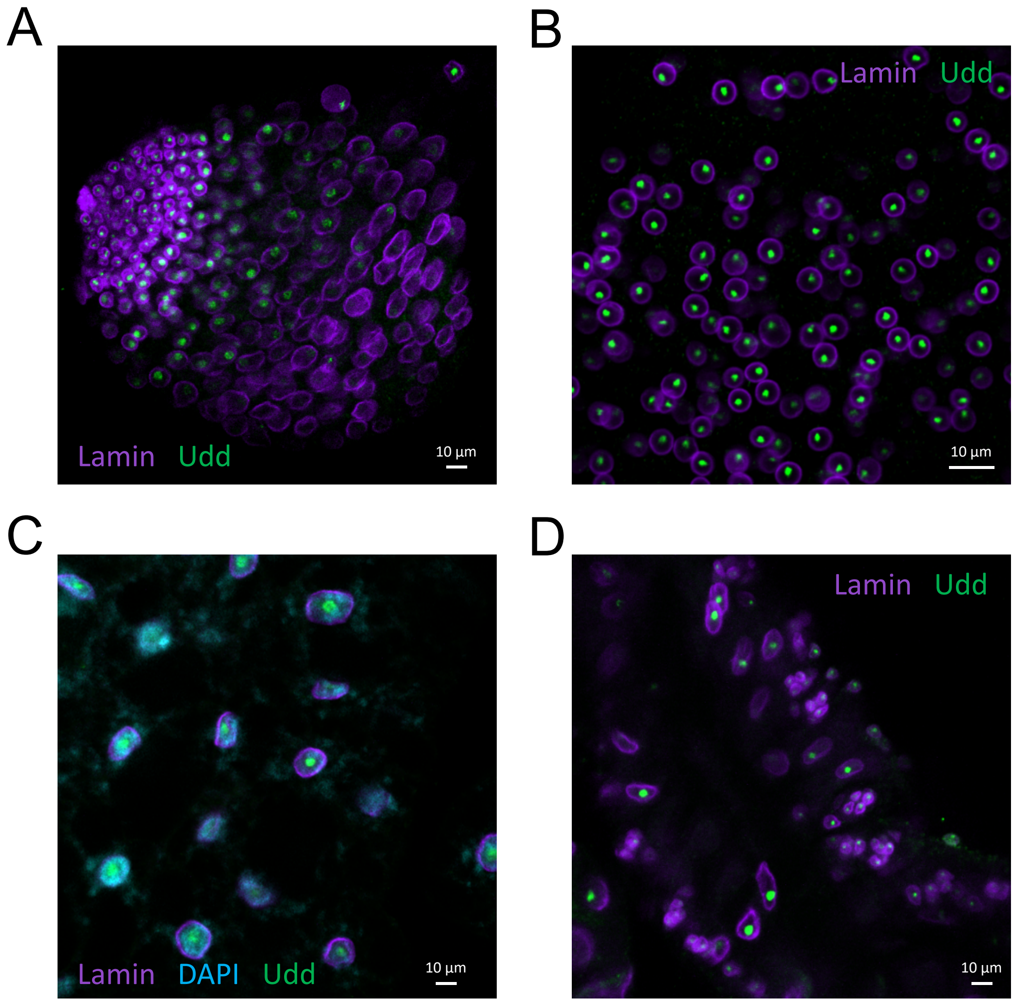

Supplementary Figure S4. Udd localization in different tissues. Immunostaining for Udd (green) and lamin (purple) showing nuclear envelope. **(A)** Larval testes. **(B)** Adult testes accessory glands. **(C)** Fat body. **(D)** Salivary glands.

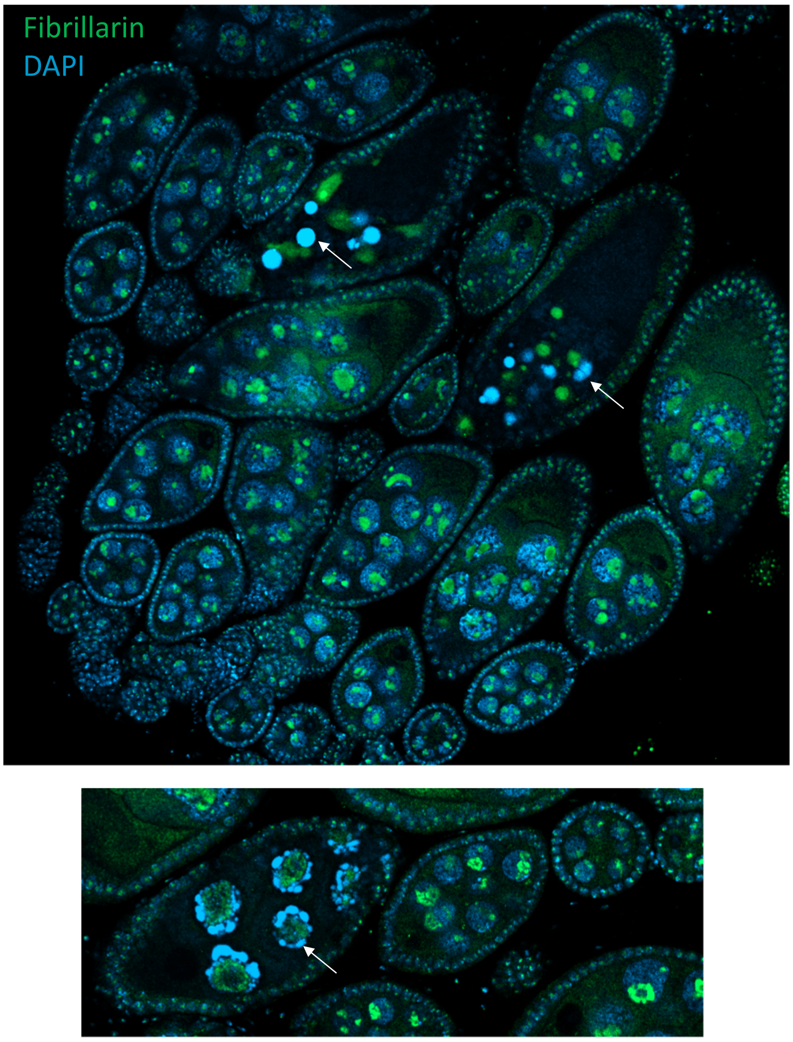

Supplementary Figure S5. Immunostaining of $udd^{0683\text{-}G4}/udd^{null}$ ovarioles for the nucleolar marker Fibrillarin (green) and DAPI. Arrows indicate examples of nurse cell nuclei with intensified DAPI staining likely as a result of apoptotic chromatin condensation at stage 11 of oogenesis (upper panel). Some ovarioles contain apoptotic nurse cells at earlier stages of oogenesis (lower panel).

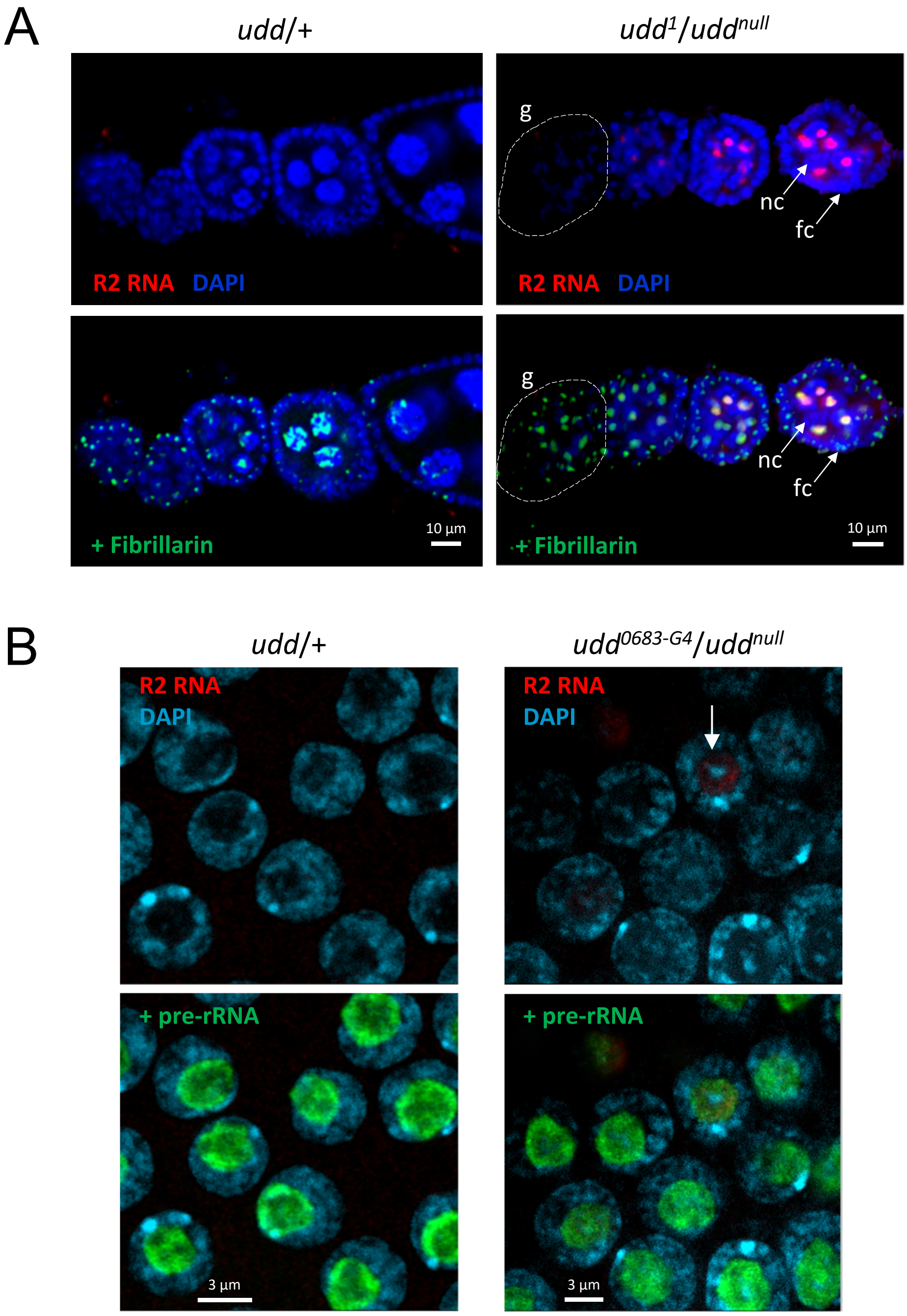

Supplementary Figure S6. (A) RNA-FISH using a probe targeting the 3'-region of the R2 transcripts (red) combined with immunostaining for nucleolar marker fibrillarin (green) in ovarioles of *udd*^{1}/*udd*^{null} mutants and control heterozygotes (*udd*/+). A germarium regions are indicated as "g" and circled with a dotted line; examples of germline nurse cells and somatic follicle cells are indicated as "nc" and "fc", respectively. FISH signal reaches a maximum in nurse cells at stages 4-5, whereas the later stages are absent in *udd*^{1}/*udd*^{null} ovaries. **(B)** Super-resolution image showing R2 and pre-rRNA smFISH in *udd*$^{0683\text{-}G4}$/*udd*^{null} and *udd*/+ follicle cells on the surface of the egg chamber**.** The nucleolus with R2 FISH signal is indicated by the white arrow.

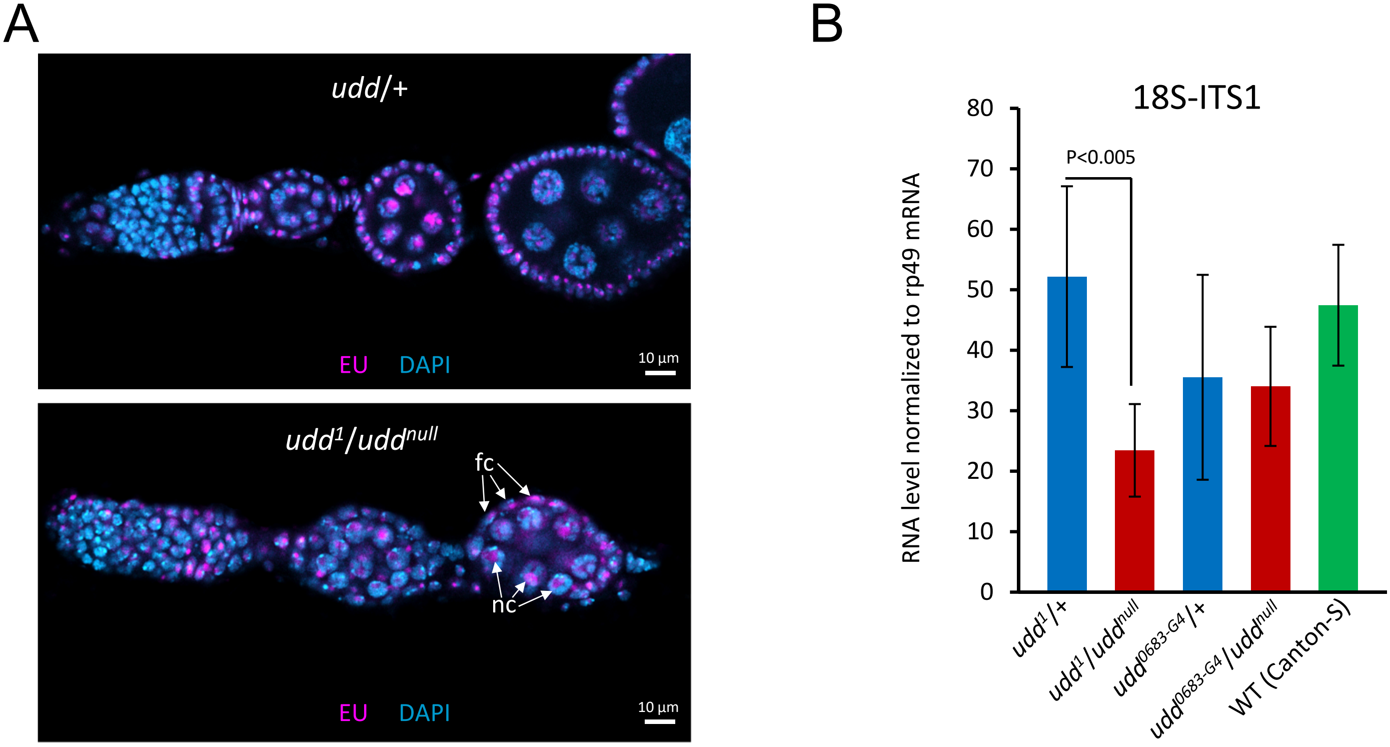

Supplementary Figure S7. Effects of Udd mutations on total nucleolar transcription in ovaries. **(A)** Detection of nascent transcription by 5-ethynyl-uridine (EU) incorporation assay in *udd*^{1}/*udd*^{null} and *udd*/+ ovarioles. Examples of germline nurse cells and somatic follicle cells are indicated as “nc” and “fc”, respectively. **(B)** RT-qPCR quantification of the total pre-rRNA level (18S-ITS1) normalized to rp49 mRNA in ovaries of *udd* mutants, control heterozygotes (*udd*/+) and wild type line Canton-S. Mean +/−s.d. and p-value based on Student’s t-test are indicated.

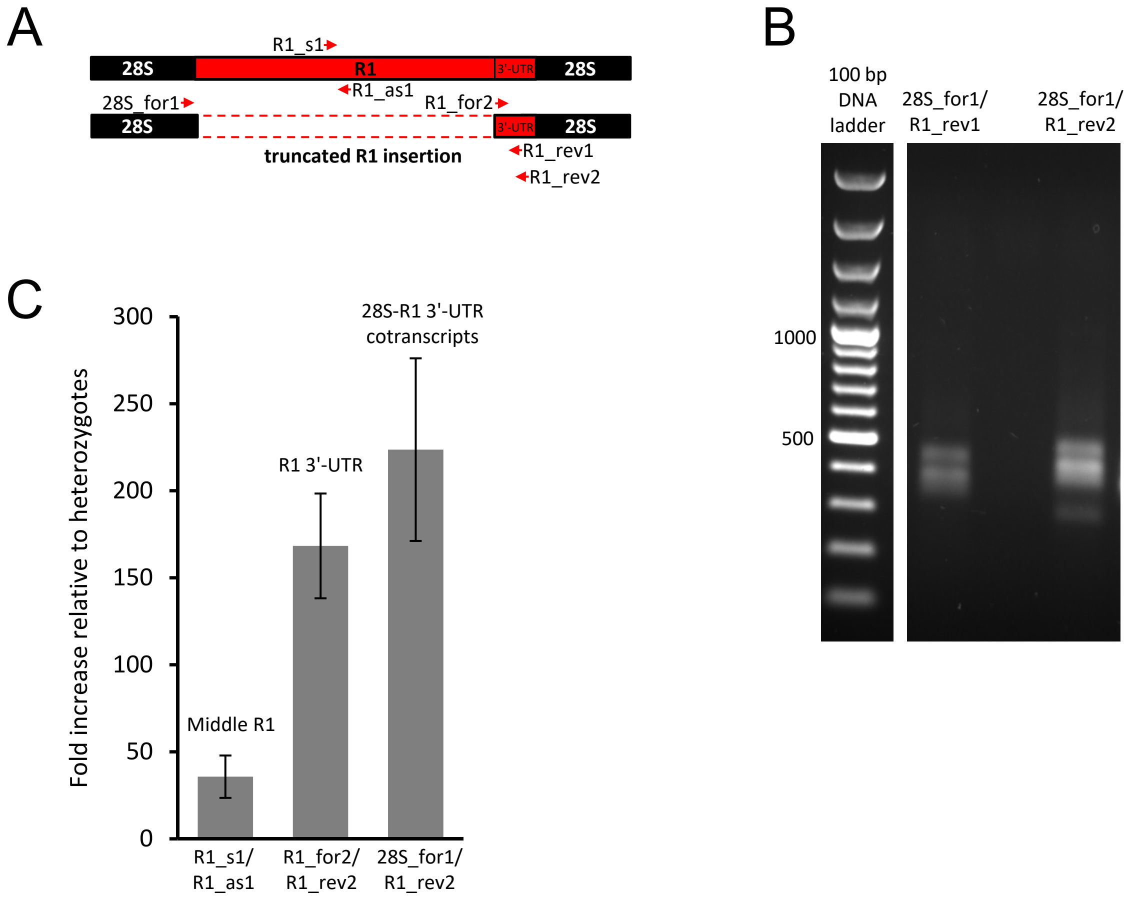

Supplementary Figure S8. The effect of Udd mutation on rDNA genes with truncated R1 insertions. **(A)** Location of PCR primers (red arrows) on the 28S gene containing R1 insertion. **(B)** Gel image of PCR-products obtained using 28S_for1/R1_rev1 and 28S_for1/R1_rev2 primer pairs detecting truncated R1 insertions (solo 3'-UTR) in the $udd^{0683\text{-}G4}/udd^{null}$ genomic DNA. 28S_for1 primer is located 243 bp upstream of the R1 insertion site. R1_rev1 and R1_rev2 are located 223 and 272 bp downstream of the beginning of the R1 3'-UTR, respectively. **(C)** RT-qPCR analysis of R1 middle region, R1 3'-UTR and 28S-R1 3'-UTR cotranscripts in $udd^{0683\text{-}G4}/udd^{null}$ ovaries. Mean fold increase +/−s.d. relative to the *udd*/+ normalized on the rp49 transcript levels are indicated. Corresponding primers are indicated below the graph.

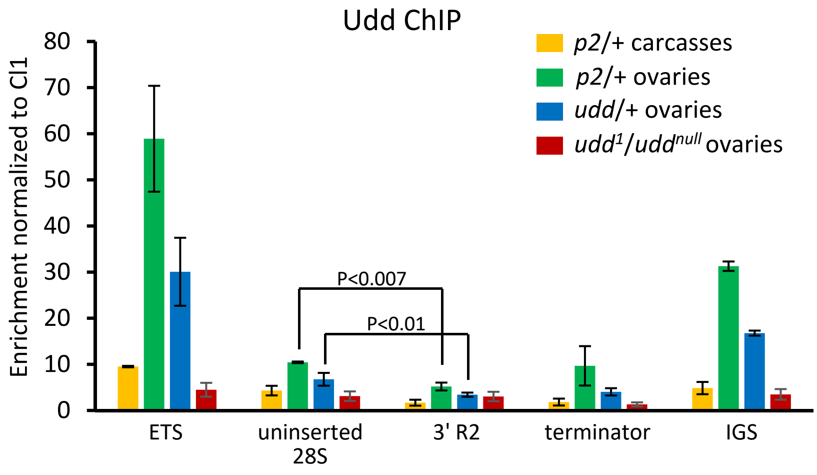

Supplementary Figure S9. Udd ChIP-qPCR analysis of ovaries and carcasses of *piwi*^{2}*/+* (*p2/+*) line and ovaries of *udd/+* and *udd*^{1}*/udd*^{null} flies. Udd binding at different regions of rDNA repeats is shown: beginning of ETS; uninserted 28S sequence; R2 insertions; the border of 28S and IGS sequences (terminator); 330bp IGS repeat (IGS). Mean +/– s.d. and p-values based on Student's t-test are indicated.

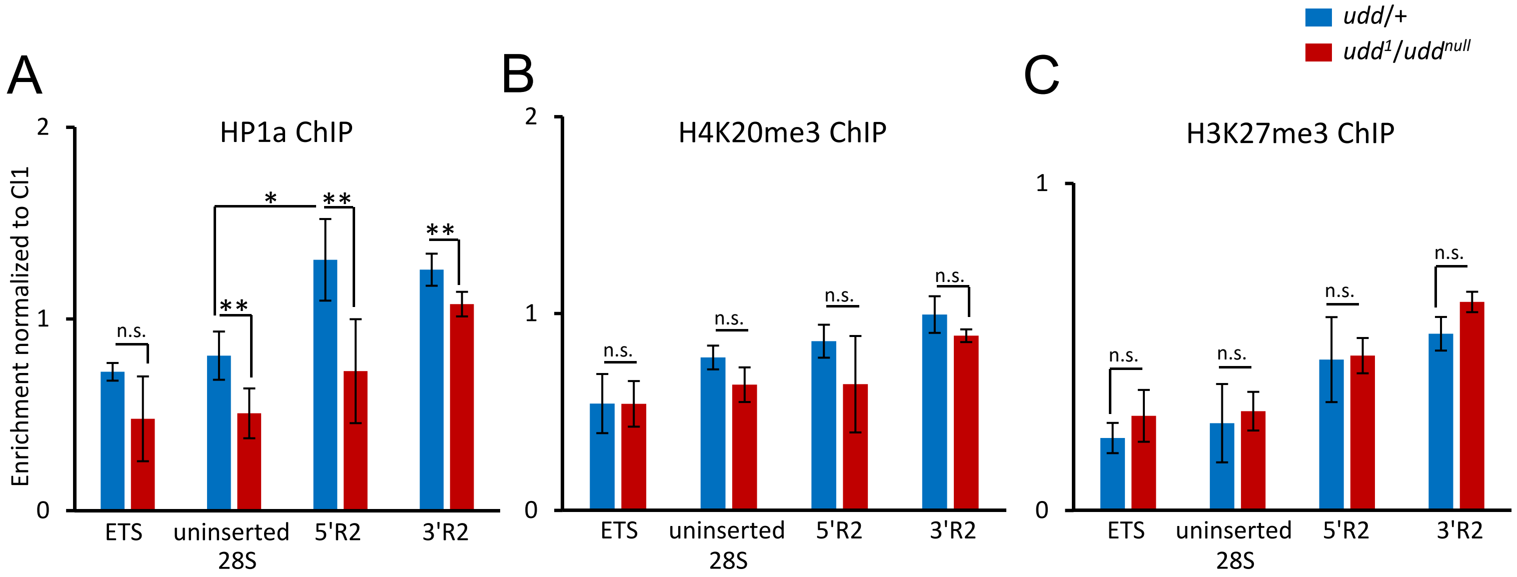

Supplementary Figure S10. ChIP-qPCR analysis of HP1a (A), and repressive histone marks H4K20me3 (B) and H3K27me3 (C) in *udd*^{1}/*udd*^{null} (red bars) and *udd*/+ (blue bars) ovaries. Mean +/− s.d. and p-values based on Student's t-test are indicated, * p<0.003, ** p<0.01, n.s. = not significant.

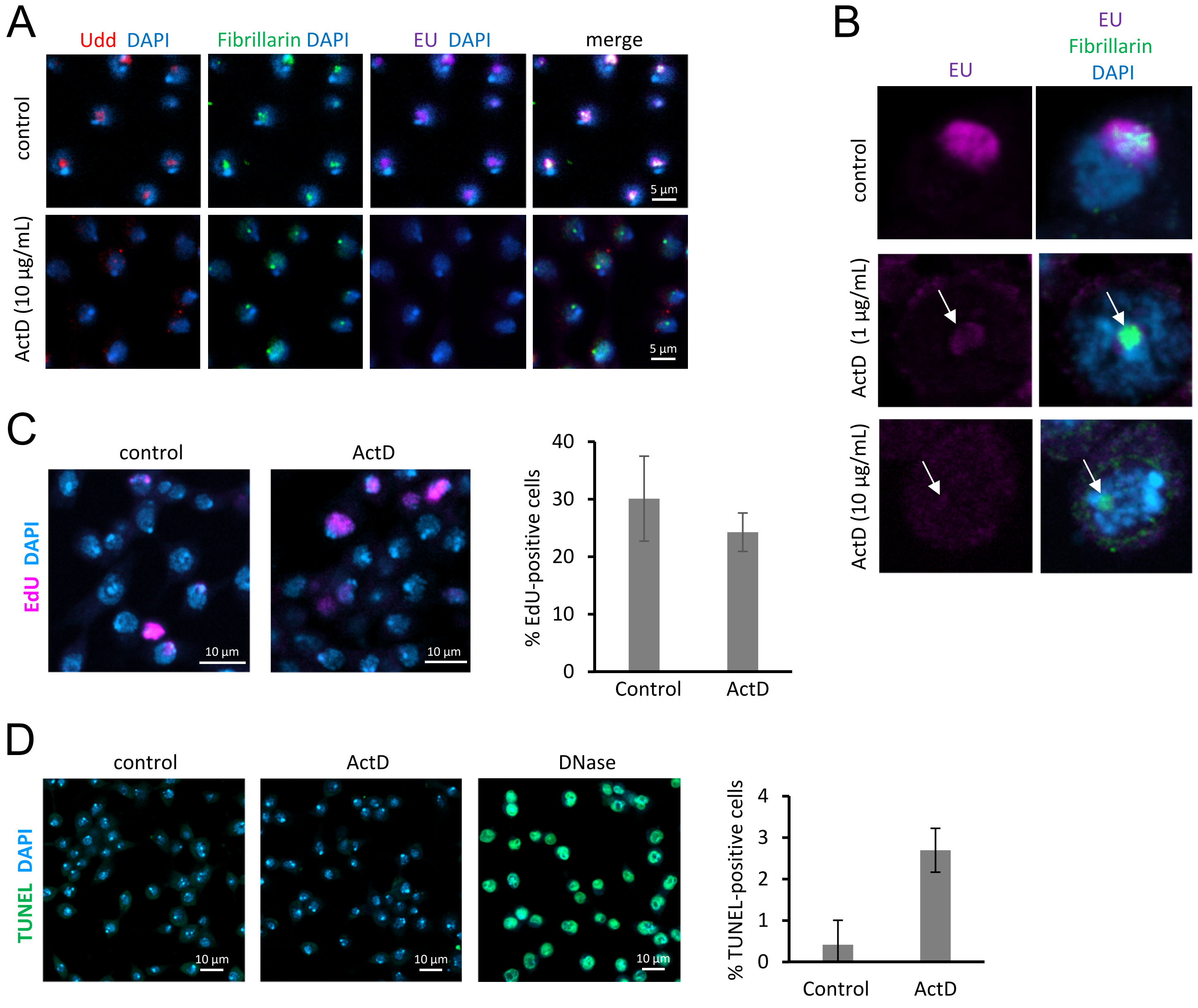

Supplementary Figure S11. (A) Control and actinomycin D (ActD)-treated OSC cells (10 µg/mL for 2 h) immunostained for Udd, fibrillarin and counterstained for nascent RNA (5-Ethynyl-uridine (EU) incorporation) and DAPI. **(B)** Fibrillarin immunostaining and the EU assay for the control and cells treated by 1 µg/mL and 10 µg/mL ActD. The nucleolar area is indicated by white arrows. **(C)** EdU incorporation assay to monitor DNA replication in ActD-treated OSC cells (10 µg/mL for 2 h). The graph shows percent of EdU-positive cells. **(D)** OSC cells stained for TUNEL (green) and DAPI (blue). After DNase treatment, TUNEL signals are detected within nuclei of all cells. Percent of TUNEL-positive cells is indicated on the graph.

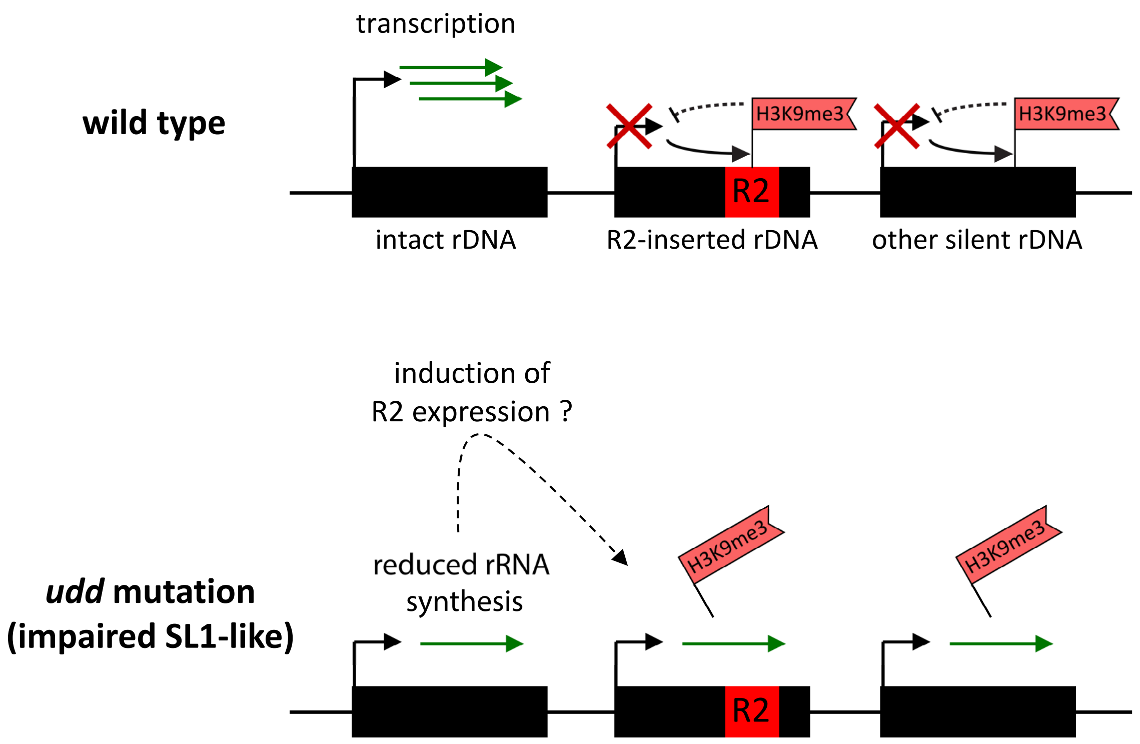

Supplementary Figure S12. A working model for the role of Sl1-like and heterochromatin components in selective regulation of rDNA repeats. Upper panel: in wild type ovarian cells, only some intact rDNA units are highly transcribed. rRNA genes with R2 insertions are less transcriptionally active that induces deposition of the H3K9me3 mark further enhancing repression. Bottom panel: the impairment of Udd or other SL1-like subunits leads to the decrease in transcription of intact rRNA genes and upregulation of normally silent rDNA units which leads to the reduction of the H3K9me3 level in their chromatin. Hypothetically, expression of R2-containing rDNA can be preferentially induced as an adaptive response to compromised rRNA synthesis.
